# A state space approach for piecewise-linear recurrent neural networks for identifying computational dynamics from neural measurements

**DOI:** 10.1371/journal.pcbi.1005542

**Published:** 2017-06-02

**Authors:** Daniel Durstewitz

**Affiliations:** Dept. of Theoretical Neuroscience, Bernstein Center for Computational Neuroscience Heidelberg-Mannheim, Central Institute of Mental Health, Medical Faculty Mannheim/ Heidelberg University, Mannheim, Germany; University of Tübingen and Max Planck Institute for Biologial Cybernetics, GERMANY

## Abstract

The computational and cognitive properties of neural systems are often thought to be implemented in terms of their (stochastic) network dynamics. Hence, recovering the system dynamics from experimentally observed neuronal time series, like multiple single-unit recordings or neuroimaging data, is an important step toward understanding its computations. Ideally, one would not only seek a (lower-dimensional) state space representation of the dynamics, but would wish to have access to its statistical properties and their generative equations for in-depth analysis. Recurrent neural networks (RNNs) are a computationally powerful and dynamically universal formal framework which has been extensively studied from both the computational and the dynamical systems perspective. Here we develop a semi-analytical maximum-likelihood estimation scheme for piecewise-linear RNNs (PLRNNs) within the statistical framework of state space models, which accounts for noise in both the underlying latent dynamics and the observation process. The Expectation-Maximization algorithm is used to infer the latent state distribution, through a global Laplace approximation, and the PLRNN parameters iteratively. After validating the procedure on toy examples, and using inference through particle filters for comparison, the approach is applied to multiple single-unit recordings from the rodent anterior cingulate cortex (ACC) obtained during performance of a classical working memory task, delayed alternation. Models estimated from kernel-smoothed spike time data were able to capture the essential computational dynamics underlying task performance, including stimulus-selective delay activity. The estimated models were rarely multi-stable, however, but rather were tuned to exhibit slow dynamics in the vicinity of a bifurcation point. In summary, the present work advances a semi-analytical (thus reasonably fast) maximum-likelihood estimation framework for PLRNNs that may enable to recover relevant aspects of the nonlinear dynamics underlying observed neuronal time series, and directly link these to computational properties.

## Author summary

Neuronal dynamics mediate between the physiological and anatomical properties of a neural system and the computations it performs, in fact may be seen as the ‘computational language’ of the brain. It is therefore of great interest to recover from experimentally recorded time series, like multiple single-unit or neuroimaging data, the underlying stochastic network dynamics and, ideally, even equations governing their statistical evolution. This is not at all a trivial enterprise, however, since neural systems are very high-dimensional, come with considerable levels of intrinsic (process) noise, are usually only partially observable, and these observations may be further corrupted by noise from measurement and preprocessing steps. The present article embeds piecewise-linear recurrent neural networks (PLRNNs) within a state space approach, a statistical estimation framework that deals with both process and observation noise. PLRNNs are computationally and dynamically powerful nonlinear systems. Their statistically principled estimation from multivariate neuronal time series thus may provide access to some essential features of the neuronal dynamics, like attractor states, generative equations, and their computational implications. The approach is exemplified on multiple single-unit recordings from the rat prefrontal cortex during working memory.

## Introduction

Stochastic neural dynamics mediate between the underlying biophysical and physiological properties of a neural system and its computational and cognitive properties (e.g. [1–4]). Hence, from a computational perspective, we are often interested in recovering the neural network dynamics of a given brain region or neural system from experimental measurements. Yet, experimentally, we commonly have access only to noisy recordings from a relatively small proportion of neurons (compared to the size of the brain area of interest), or to lumped surface signals like local field potentials or the EEG. Inferring from these the computationally relevant dynamics is therefore not trivial, especially since both the recorded signals (e.g., spike sorting errors; [5]) as well as the neural system dynamics itself (e.g., stochastic synaptic release; [6]) come with a good deal of noise. The stochastic nature of neural dynamics has, in fact, been deemed crucial for perceptual inference and decision making [7–9], and potentially helps to avoid local minima in task learning or problem solving [10].

Speaking in statistical terms, 'model-free' techniques which combine delay embedding methods with nonlinear basis expansions and kernel techniques have been one approach to the problem [11; 12]. These techniques provide informative lower-dimensional visualizations of population trajectories and (local) approximations to the neural flow field, but they may highlight only certain, salient aspects of the dynamics (but see [13]) and, in any case, do not directly return distribution generating equations or underlying computations. Alternatively, state space models, a statistical framework particularly popular in engineering and ecology (e.g. [14]), have been adapted to extract lower-dimensional, probabilistic neural trajectory flows from higher-dimensional recordings [15–25]. State space models link a process model of the unobserved (latent) underlying dynamics to the experimentally observed time series via observation equations, and differentiate between stochasticity in the process and observation noise (e.g. [26]). So far, with few exceptions (e.g. [23; 27]), these models assumed linear latent dynamics, however. Although this may often be sufficient to yield lower-dimensional smoothed trajectories, it implies that the recovered dynamical model may be less apt for capturing highly nonlinear dynamical phenomena in the observations, and will by itself not be powerful enough to reproduce a range of important dynamical and computational processes in the nervous system, among them multi-stability which has been proposed to underlie neural activity during working memory [28–32], limit cycles (stable oscillations), or chaos (e.g. [33]).

Here we derive a new state space algorithm based on piecewise-linear (PL) recurrent neural networks (RNN). It has been shown that RNNs with nonlinear activation functions can, in principle, approximate any dynamical system's trajectory or, in fact, dynamical system itself (given some general conditions; [34–36]). Thus, in theory, they are powerful enough to recover whatever dynamical system is underlying the experimentally observed time series. Piecewise linear activation functions, in particular, are by now the most popular choice in deep learning algorithms [37–39], and considerably simplify some of the derivations within the state space framework (as shown later). They may also be more apt for producing working memory-type activity with longer delays if for some units the transfer function happens to coincide with the bisectrix (cf. [40]), and ease the analysis of fixed points and stability. We then apply this newly derived algorithm to multiple single-unit recordings from the rat prefrontal cortex obtained during a classical delayed alternation working memory task [41].

## Results

### State space model

This article considers simple discrete-time piecewise-linear (PL) recurrent neural networks (RNN) of the form

$$z_{t}=Az_{t-1}+W\;\max\{0,z_{t-1}-\theta \}+Cs_{t}+\varepsilon_{t}\;,\varepsilon_{t}∼N(0,\Sigma ),$$ (1)

where **z**_*t*_ = (*z*_1*t*_…*z*_*Mt*_)^*T*^ is the (*M*×1)-dimensional (latent) neural state vector at time *t* = 1…*T*, **A** = *diag*([*a*_11_…*a*_*MM*_]) is an *M*×*M* diagonal matrix of auto-regression weights, **W** = (0 *w*_12_…*w*_1*M*_, *w*_21_ 0 *w*_23_…*w*_2*M*_, *w*_31_ *w*_32_ 0 *w*_34_…*w*_3*M*_,…) is an *M*×*M off*-diagonal matrix of connection weights, **θ** = (*θ*_1_…*θ*_*M*_)^*T*^ is a set of (constant) activation thresholds, **s**_*t*_ is a sequence of (known) external *K*-dimensional inputs, weighted by (*M*×*K*) matrix **C**, and **ε**_*t*_ denotes a Gaussian white noise process with diagonal covariance matrix $\Sigma =diag([\sigma_{11}^{2}\ldots \sigma_{MM}^{2}])$. The max-operator is assumed to work element-wise.

In physiological terms, latent variables *z*_*mt*_ are often interpreted as a membrane potential (or current) which gives rise to spiking activity as soon as firing threshold *θ*_*m*_ is exceeded (e.g. [42,43]). According to this interpretation, the diagonal elements in **A** may be seen as the neurons’ individual membrane time constants, while the off-diagonal elements in **W** represent the between-neuron synaptic connections which multiply with the presynaptic firing rates. In statistical terms, (1) has the form of an auto-regressive model with a nonlinear basis expansion in variables *z*_*mt*_ (e.g. [44;45]), which retains linearity in parameters **W** for ease of estimation. Restricting model parameters, e.g. **Σ**, to be of diagonal form, is common in such models to avoid over-specification and help identifiabiliy (e.g. [26; 46]; see also further below). For instance, including a diagonal in **W** would be partly redundant to parameters **A** (strictly so in a pure linear model). For similar reasons, and for ease of presentation, in the following we will focus on a model for which *K* = *M* and **C** = **I** (i.e., no separate scaling of the inputs), although the full model as stated above, Eq 1, was implemented as well (and code for it is provided; of course, the case *K*>*M* could always be accommodated by pre-multiplying **s**_*t*_ by some *predefined* matrix **C**, obtained e.g. by PCA on the input space). While different model formulations are around in the computational neuroscience and machine learning literature, often they may be related by a simple transformation of variables (see [47]) and, as long as the model is powerful enough to express the whole spectrum of basic dynamical phenomena, details of model specification may also not be overly crucial for the present purposes.

A particular advantage of the PLRNN model is that all its fixed points can be obtained easily analytically by solving (in the absence of external input) the 2^*M*^ linear equations

$$z_{*}={\left(A+W_{\Omega }-I\right)}^{-1}W_{\Omega }\theta ,$$ (2)

where Ω is to denote the set of indices of units for which we assume *z*_*m*_ ≤ *θ*_*m*_, and **W**_Ω_ the respective connectivity matrix in which all columns from **W** corresponding to units in Ω are set to 0. Obviously, to make z_*_ a true fixed point of (1), the solution to (2) has to be consistent with the defined set Ω, that is z_*m_ ≤ *θ*_*m*_ has to hold for all *m* ∈ Ω and z_*m_ > *θ*_*m*_ for all *m* ∉ Ω. For networks of moderate size (say *M*<30) it is thus computationally feasible to explicitly check for all fixed points and their stability.

For estimation from experimental data, latent state model (1) is then connected to some *N*-dimensional observed vector time series **X** = {**x**_t_} via a simple linear-Gaussian model,

$$x_{t}=B\phi (z_{t})+\eta_{t}\;,\eta_{t}∼N(0,\Gamma ),$$ (3)

where *ϕ*(**z**_*t*_) ≔ max{**0**,**z**_*t*_−**θ**}, {**η**_*t*_} is the (white Gaussian) observation noise series with diagonal covariance matrix $\Gamma =diag([\gamma_{11}^{2}\ldots \gamma_{NN}^{2}])$, and **B** an *N*×*M* matrix of regression weights. Thus, the idea is that only the PL-transformed activation *ϕ*(**z**_*t*_) reaches the ‘observation surface’ as, e.g., with spiking activity when the underlying membrane dynamics itself is not visible. We further assume for the initial state,

$$z_{1}∼N(\mu_{0}+s_{1},\Sigma ),$$ (4)

which has, for simplicity, the same covariance matrix as the process noise in general (reducing the number of to be estimated parameters). In the case of multiple, temporally separated trials, we allow each one to have its own individual initial condition **μ**_*k*_, *k* = 1…*K*.

The general goal here is to determine both the model’s unknown parameters **Ξ** = {**μ**_0_,**A**,**W**,**Σ**,**B**,**Γ**} (assuming fixed thresholds **θ** for now) as well as the unobserved, latent state path **Z** ≔ {**z**_*t*_} (and its second-order moments) from the experimentally observed time series {**x**_t_}. These could be, for instance, properly transformed multivariate spike time series or neuroimaging data. This is accomplished here by the Expectation-Maximization (EM) algorithm which iterates state (E) and parameter (M) estimation steps and is developed in detail for model (1) and (3) in the Methods. In the following I will first discuss state and parameter estimation separately for the PLRNN, before describing results from the full EM algorithm in subsequent sections. This will be done along two toy problems, a higher-order nonlinear oscillation (stable limit cycle), and a simple 'working memory' paradigm in which one of two discrete stimuli had to be retained across a temporal interval. Finally, the application of the validated PLRNN EM algorithm will be demonstrated on multiple single-unit recordings obtained from rats on a standard working memory task (delayed alternation; data from [41], kindly provided by Dr. James Hyman, University of Nevada, Las Vegas).

### State estimation

The latent state distribution, as explained in Methods, is a high-dimensional (piecewise) Gaussian mixture with the number of components growing as 2^T×M^ with sequence length T and number of latent states M. Here a semi-analytical, approximate approach was developed that treats state estimation as a combinatorial problem by first searching for the mode of the full distribution (cf. [16; 48]; in contrast, e.g., to a recursive filtering-smoothing scheme that makes local (linear-Gaussian) approximations, e.g. [15; 26]). This approach amounts to solving a high (2^M×T^)-dimensional piecewise linear problem (due to the piecewise quadratic, in the states **Z**, log-likelihood Eqs 6 and 7). Here this was accomplished by alternating between (1) solving the linear set of equations implied by a given set of linear constraints **Ω** ≔ {(*m*,*t*)|*z*_*mt*_ ≤ *θ*_*m*_} (cf. Eq 7 in Methods) and (2) flipping the sign of the constraints violated by the current solution z_*_(Ω) to the linear equations, thus following a path through the (M×T)-dimensional binary space of linear constraints using Newton-type iterations (similar as in [49], see Methods; note that here the ‘constraints’ are not fixed as in quadratic programming problems). Given the mode and state covariance matrix (evaluated at the mode from the negative inverse Hessian), all other expectations needed for the EM algorithm were then derived analytically, with one exception that was approximated (see Methods for full details).

**Fig 1:**
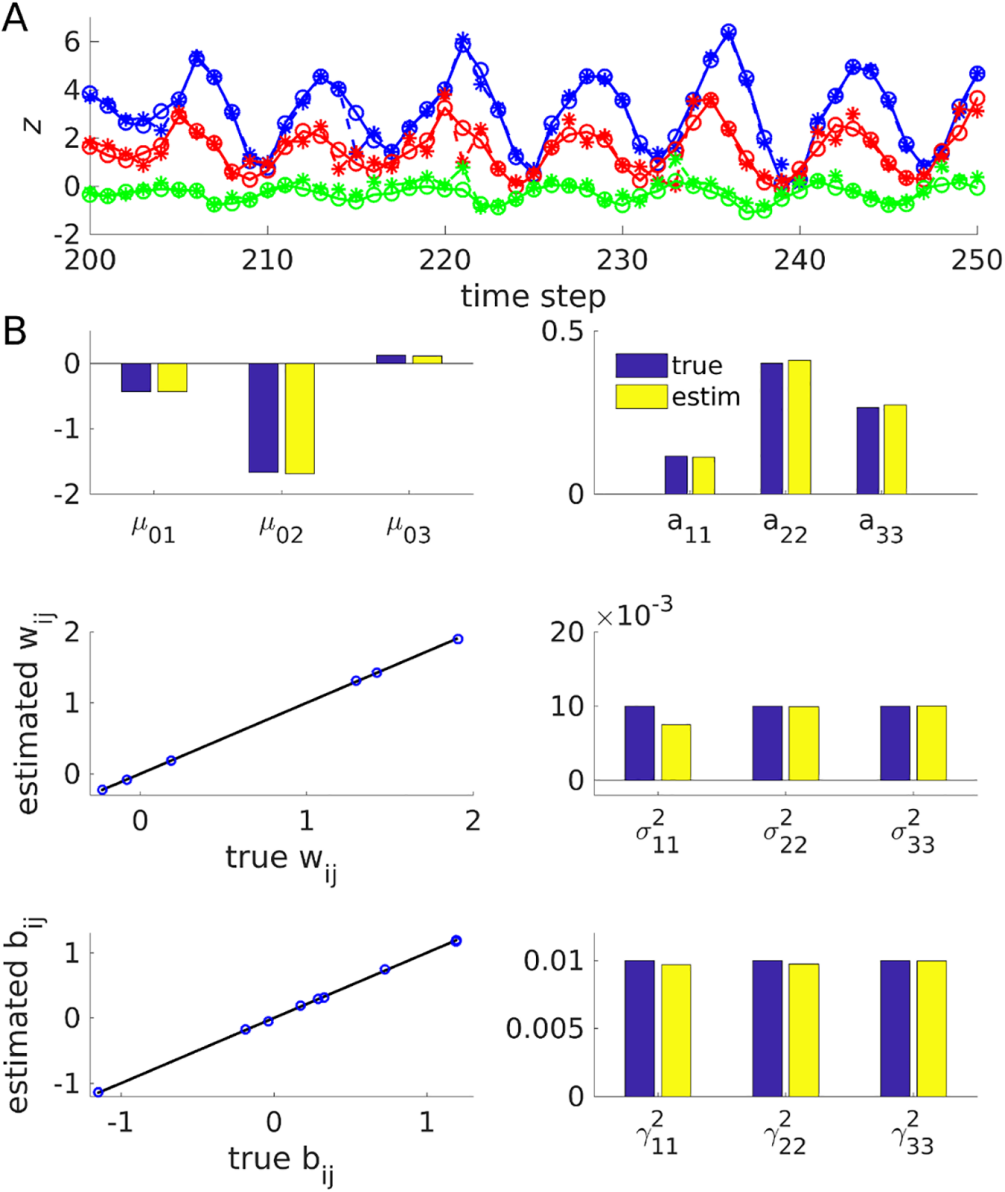
State and parameter estimates for nonlinear cycle example. (A) True (solid/ open-circle lines) and estimated (dashed-star lines) states over some periods of the simulated limit cycle generated by a 3-state PLRNN when true parameters were provided (for this example, **θ** ≈ (0.86,0.09,–0.85); all other parameters as in B, see also provided Matlab file ‘PLRNNoscParam.mat’). ‘True states’ refers to the actual states from which the observations **X** were generated. Inputs of *s*_*it*_ = 1 were provided to units *i* = 1 and *i* = 2 on time steps 1 and 10 of each cycle, respectively. Note that true and inferred states are tightly overlapping in this low-noise example (such that the ‘stars’ appear on top of the ‘open circles’). (B) True and estimated model parameters for (from top-left to bottom-right) **μ**_0_,**A**,**W**,**Σ**,**B**,**Γ**, when true states (but not their higher-order moments) were provided. Bisectrix lines (black) indicate identity.

The toy problems introduced above were used to assess the quality of these approximations. For the first toy problem, an order-15 limit cycle was produced with a PLRNN consisting of three recurrently coupled units, inputs to units #1 and #2, and parameter settings as indicated in Fig 1 and provided Matlab file ‘PLRNNoscParam’. The limit cycle was repeated for 50 full cycles (giving 750 data points) and corrupted by process noise (cf. Fig 1). These noisy states (arranged in a (3 x 750) matrix **Z**) were then transformed into a (3 x 750) output matrix **X**, to which observation noise was added, through a randomly drawn (3 x 3) regression weight matrix **B**. State estimation was started from a random initial condition. True (but noise-corrupted) and estimated states for this particular problem are illustrated in Fig 1A, indicating a tight fit (although some fraction of the linear constraints were still violated, ≈0.27% in the present example and <2.3% in the working memory example below; see Methods on this issue).

**Fig 2:**
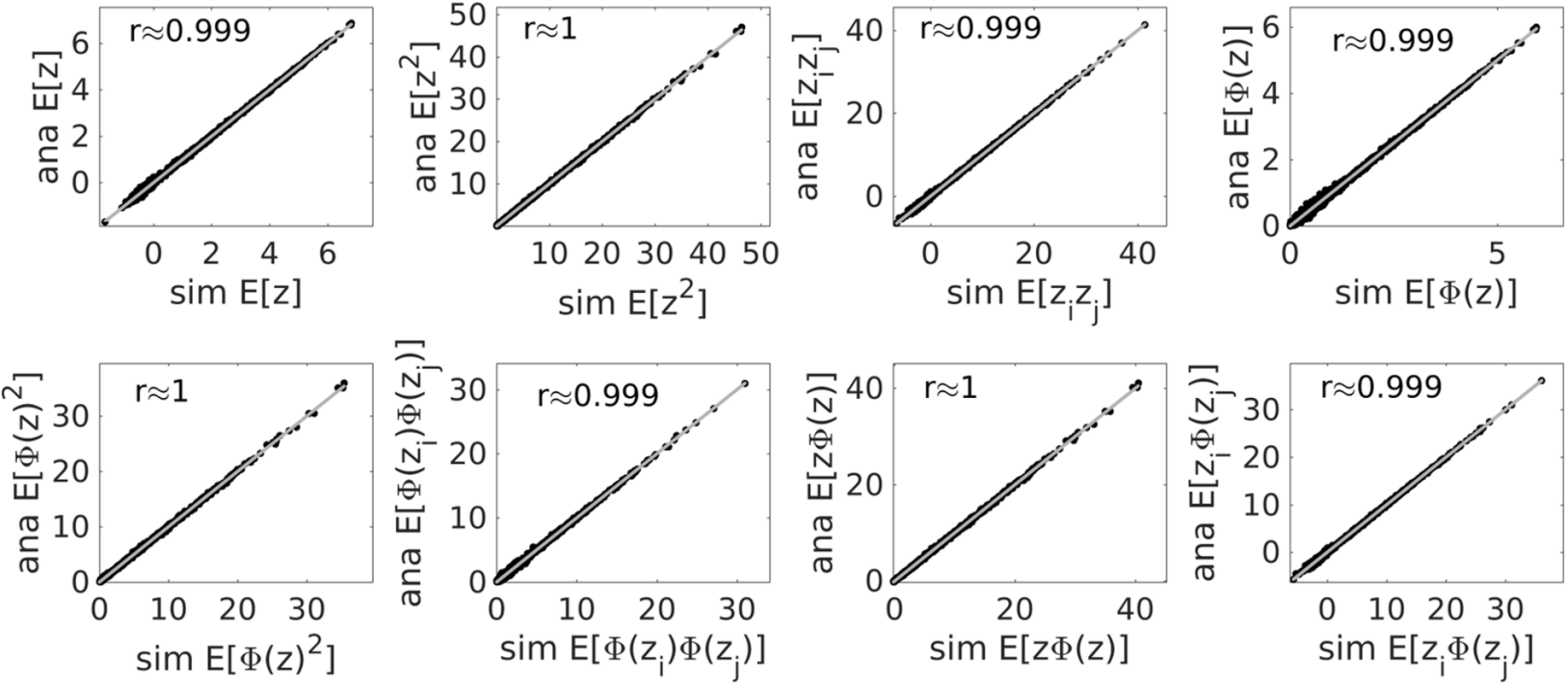
Agreement between simulated (*x*-axes) and semi-analytical (*y*-axes) solutions for state expectancies for the model from Fig 1 across all three state variables and *T* = 750 time steps. Here, *ϕ*(*z*_*i*_) ≔ max{0,*z*_*i*_−*θ*_*i*_} is the PL activation function. Simulated state paths and their moments were generated using a bootstrap particle filter with 10^4^ particles. Bisectrix lines in gray indicate identity.

To examine more systematically the quality of the approximate-analytical estimates of the first and second order moments of the joint distribution across states *z* and their piecewise linear transformations *ϕ*(*z*), samples from p(**Z**|**X**) were simulated using bootstrap particle filtering (see Methods). Although these simulated samples are based only on the filtering (not the smoothing) steps (and (re-)sampling schemes may have issues of their own; e.g. [26], analytical and sampling estimates were in tight agreement, correlating almost to 1 for this example, as shown in Fig 2.

**Fig 3:**
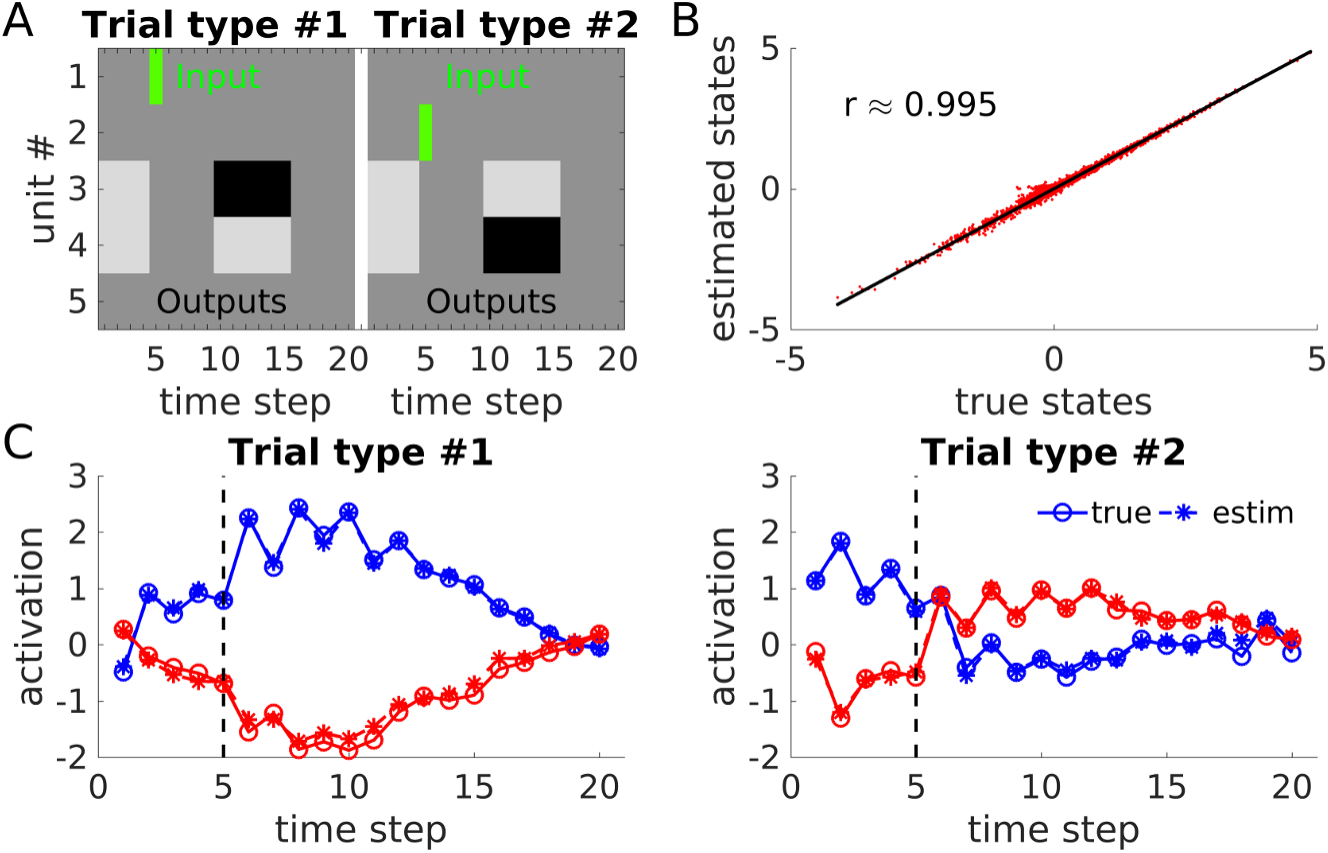
State estimation for ‘working memory’ example when true parameters were provided. (A) Setup of the simulated working memory task: Stimulus inputs (green bars, *s*_*it*_ = 1, and 0 otherwise) and requested outputs (black = 1, light-gray = 0, dark-grey = no output required) across the 20 time points of a working memory trial (with two different trial types) for the 5 PLRNN units. (B) Correlation between estimated and true states (i.e., those from which the observations **X** were generated) across all five state variables and *T* = 800 time steps. Bisectrix in black. (C) True (open-circle/ solid lines) and estimated (star-dashed lines) states for output units #3 (blue) and #4 (red) when *s*_15_ = 1 (left) or *s*_25_ = 1 (right) for single example trials. Note that true and inferred states are tightly overlapping in this low-noise example (such that the ‘stars’ often appear on top of the ‘open circles’). Although working memory PLRNNs may, in principle, be explicitly designed (see text), here a 5-state PLRNN was first trained by conventional gradient descent (real-time recurrent-learning [50]) to perform the task in A, to yield more ‘natural’ and less uniform ground truth states and parameters. Here, all *θ*_*i*_ = 0 (implying that there can only be one stable fixed point). See Matlab file ‘PLRNNwmParam.mat’ and Fig 4 for details on parameters.

**Fig 4:**
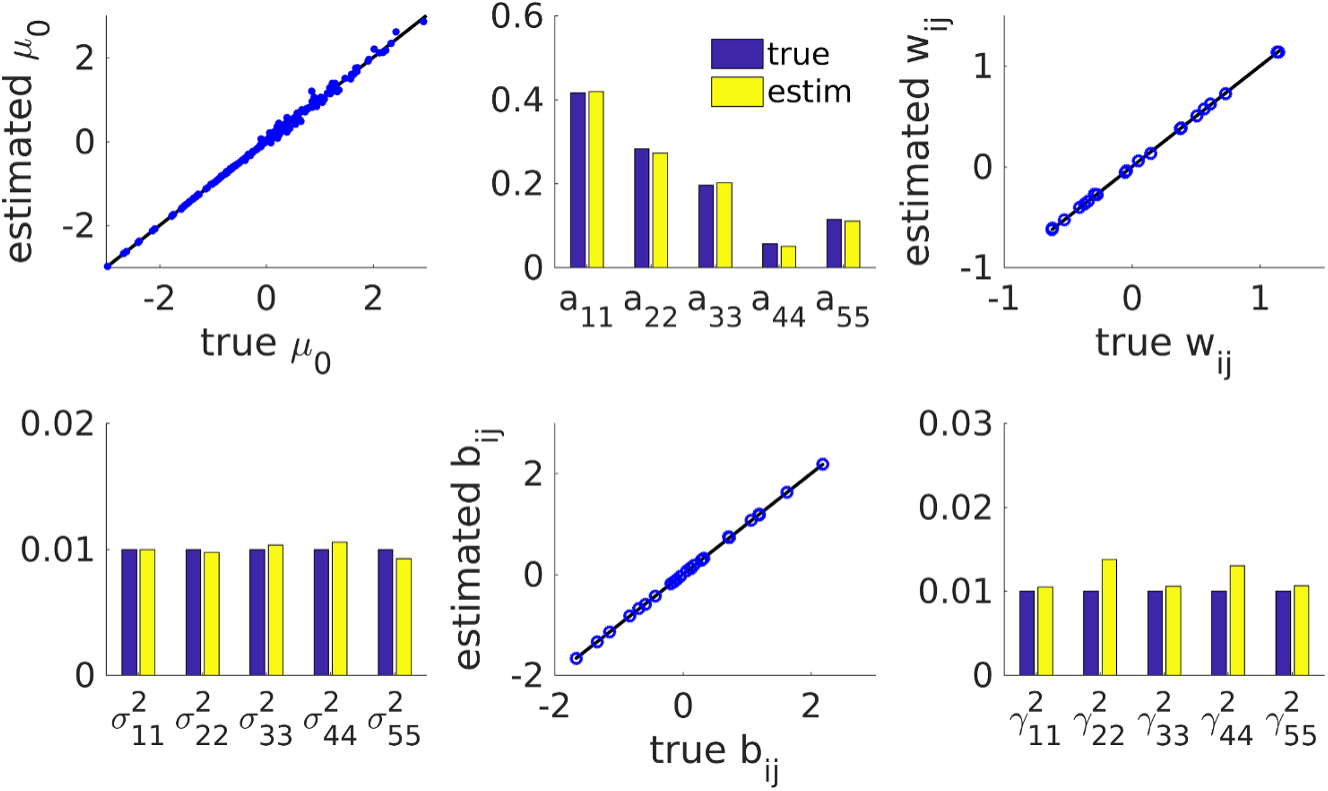
True and estimated parameters for the working memory PLRNN (cf. Fig 3) when true states were provided. From top-left to bottom-right, estimates for: **μ**_0_,**A**,**W**,**Σ**,**B**,**Γ**. Note that most parameter estimates were highly accurate, although all state covariance matrices still had to be estimated as well (i.e., with the true states provided as initialization for the E-step). Bisectrix lines in black indicate identity.

Fig 3A illustrates the setup of the ‘two-cue working memory task’, chosen for later comparability with the experimental setup. A 5-unit PLRNN was first trained by conventional gradient descent (‘real-time recurrent learning’ (RTRL), see [50; 51]) to produce a series of six 1’s on unit #3 and six 0’s on unit #4 five time steps after an input (of 1) occurred on unit #1, and the reverse pattern (six 0’s on unit #3 and six 1’s on unit #4) five time steps after an input occurred on unit #2. A stable PLRNN with a reasonable solution to this problem was then chosen for further testing the present algorithm (cf. Fig 3C). (While the RTRL approach was chosen to derive a working memory circuit with reasonably ‘realistic’ characteristics like a wider distribution of weights, it is noted that a multi-stable network is relatively straightforward to construct explicitly given the analytical accessibility of fixed points (see Methods); for instance, choosing **θ** = (0.5 0.5 0.5 0.5 2), **A** = (0.9 0.9 0.9 0.9 0.5), and **W** = (0 *ω* − *ω* − *ω* − *ω*, *ω* 0 − *ω* − *ω* – *ω*, − *ω* − *ω* 0 *ω* – *ω*, − *ω* − *ω ω* 0 − *ω*, 11110) with *ω* = 0.2, yields a tri-stable system.) Like for the limit cycle problem before, the number of observations was taken to be equal to the number of latent states, and process and observation noise were added (see Fig 4 and Matlab file ‘PLRNNwmParam’ for specification of parameters). The system was simulated for 20 repetitions of each trial type (i.e., cue-1 or cue-2 presentations) with different noise realizations and each ‘trial’ started from its own initial condition **μ**_*k*_ (see Methods), resulting in a total series length of T = 20×2×20 = 800 (although, importantly, in this case the time series consisted of distinct, temporally segregated trials, instead of one continuous series, and was treated as such an ensemble of series by the algorithm). As before, state estimation started from random initial conditions and was provided with the correct parameters, as well as with the observation matrix **X**. While Fig 3B illustrates the correlation between true (i.e., simulated) and estimated states across all trials and units, Fig 3C shows true and estimated states for a representative cue-1 (left) and cue-2 (right) trial, respectively. Again, our procedure for obtaining (or approximating) the maximum a-posteriori (MAP) estimate of the state distribution appears to work quite well (in general, only locally optimal or approximate solutions may be achieved, however, and the algorithm may have to be repeated with different state initializations; see Methods).

### Parameter estimation

Given the true states, how well would the algorithm retrieve the parameters of the PLRNN? To assess this, the actual model states (which generated the observations **X**) from simulation runs of the oscillation and the working memory task described above were provided as initialization for the E-step. Based on these, the algorithm first estimated the state covariances for *z* and *ϕ*(*z*) (see above), and then the parameters in a second step (i.e., the M-step). Note that the parameters can all be computed analytically given the state distribution (see Methods), and, provided the state covariance matrices (summed across time) as required in Eq 17A, 17D and 17F are non-singular, have a unique solution. Hence, in this case, any misalignment with the true model parameters can only come from one of two sources: i) estimation was based on one finite-length noisy realization of the PLRNN process, ii) all *second order moments* of the state distribution were still *estimated* based on the true state vectors. However, as can be appreciated from Fig 1B (oscillation) and Fig 4 (working memory), for the two (relatively low-noise) example scenarios studied here, all parameter estimates still agreed tightly with those describing the true underlying model.

In the more general case where *both* the states and the parameters are unknown and only the observations are given, note that the model as stated in Eqs 1 & 3 is over-specified as, for instance, at the level of the observations, additional variance placed into Σ may be compensated for by adjusting Γ accordingly, and by rescaling **W** and, within limits, **A** (cf. [52; 53]). In the following we therefore always arbitrarily fixed **Σ** (to some scalar; see Methods), as common in many latent variable models (like factor analysis), including state space models (e.g. [27; 46]). It may be worth noting here that the relative size of **Σ** vs. **Γ** determines how much weight is put on temporal consistency among states (“**Σ**<**Γ**”) vs. fitting of the observations (“**Σ**>**Γ**”) within the likelihood, Eq 5.

### Joint estimation of states and parameters by EM

The observations above confirm that our algorithm finds satisfactory approximations to the underlying state path and state covariances when started with the right parameters, and—vice versa—identifies the correct parameters when provided with the true states. Indeed, the M-step, since it is exact, can only increase the expected log-likelihood Eq 5 with the present state expectancies fixed. However, due to the system's piecewise-defined discrete nature, modifying the parameters may lead to a new set of constraint violations, that is may throw the system into a completely different linear subspace which may imply a decrease in the likelihood in the E-step. It is thus not guaranteed that a straightforward EM algorithm converges (cf. [54; 55]), or that the likelihood would even monotonically increase with each EM iteration.

**Fig 5:**
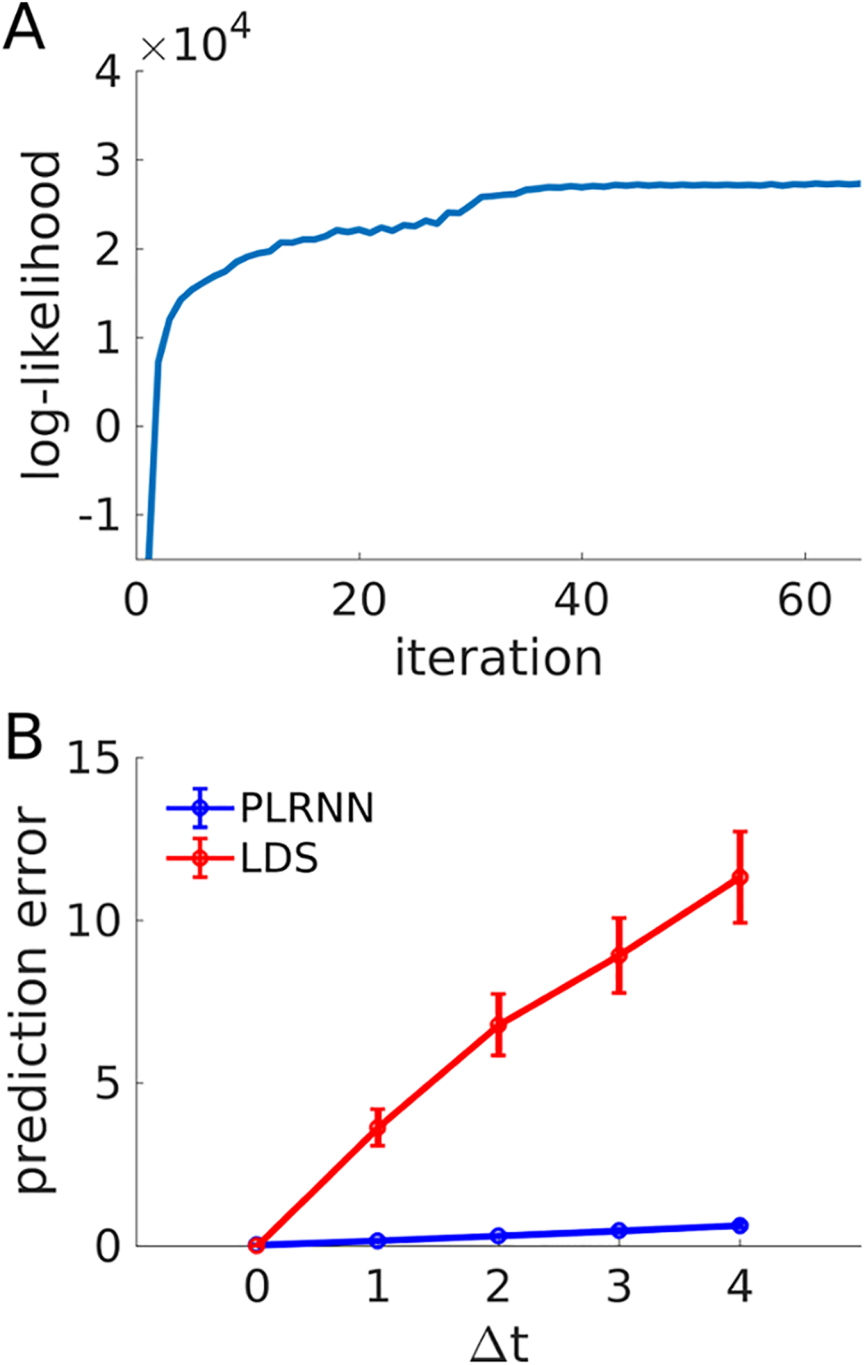
Performance of full EM algorithm on working memory model. (A) Log-likelihood as a function of EM iteration for the highest-likelihood run out of all 240 initializations. As in this example, the log-likelihood, although generally increasing, was not always monotonic (note the little ripples; see discussion in Results). (B) Mean squared prediction error, $\mathrm{avg}[{\left(x_{it}-{\hat{x}}_{it}\right)}^{2}]$, between true ($\left\{x_{t}\right\}$) and predicted ($\left\{{\overset{⌢}{x}}_{t}\right\}$) observations across all 20 output variables and the 5 highest-likelihood solutions, as a function of ahead-prediction time step Δ*t*, for the original PLRNN (blue curve) and for a linear dynamical system (LDS; red curve) estimated via EM from the same, PLRNN-generated data. Note that while the true and estimated observations agree almost perfectly for both the PLRNN and LDS if predicted directly from the inferred states (i.e., ${\overset{⌢}{x}}_{t}=B\phi ({\hat{z}}_{t})$), prediction quality severely decays for the LDS while remaining high for the PLRNN if $\left\{{\overset{⌢}{x}}_{t}\right\}$-predictions were made from states forecast Δ*t* time steps into the future (see text for further explanation; note that a slight decay in prediction quality across Δ*t* is inevitable because of the process noise). Error bars = SEM.

**Fig 6:**
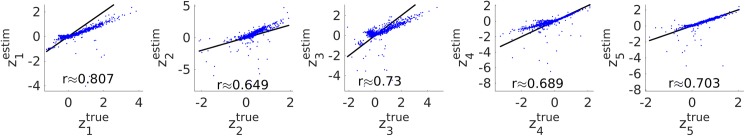
State estimates for ML solution (cf. Fig 5) from the full EM algorithm on the working memory model. In this example, true and estimated states were nicely linearly related, although mostly with regression slopes deviating from 1 (see text for further discussion). State estimation in this case was performed by inverting only the single constraint corresponding to the largest deviation on each iteration (see Methods). Bisectrix lines in black indicate identity.

To examine this issue, full EM estimation of the WM model (as specified in Fig 4, using *N* = 20 outputs in this case) was run 240 times, starting from different random, uniformly distributed initializations for the parameters. Fig 5B (Δ*t* = 0) gives, for the five highest likelihood solutions across all 240 runs (Fig 5A), the mean squared error (MSE) $\mathrm{avg}[{\left(x_{it}-{\hat{x}}_{it}\right)}^{2}]$ between actual neural observations *x*_*it*_ and model predictions ${\hat{x}}_{it}$, which is close to 0 (and, correspondingly, correlations between predicted and actual observations were close to 1). With respect to the inferred states, note that estimated and true model states may not be in the same order, as any permutation of the latent state indices together with the respective columns of observation matrix **B** will be equally consistent with the data **X** (see also [27]). For the WM model examined here, however, partial order information is implicitly provided to the EM algorithm through the definition of unit-specific inputs *s*_*it*_. For the present example, true and estimated states for the highest likelihood solution were nicely linearly correlated for all 5 latent variables (Fig 6), but some of the regression slopes significantly differed from 1, indicating a degree of freedom in the scaling of the states. Note that if the system were strictly linear, the states would be identifiable only up to a linear transformation in general, since any multiplication of the latent states by some matrix **V** could essentially be reversed at the level of the outputs by back-multiplying **B** with **V**^-1^ (cf. [27]). Likewise, in the present *piecewise linear* system, one may expect that there is a class of piecewise-linear transformations of the states which is still compatible with the observed outputs, and hence that the model is only identifiable up to this class of transformations (a general issue with state space models, of course, not particular to the present one; cf. [53]). However, this might not be a too serious issue, if one is primarily interested in the latent dynamics (rather than in the exact parameters).

**Fig 7:**
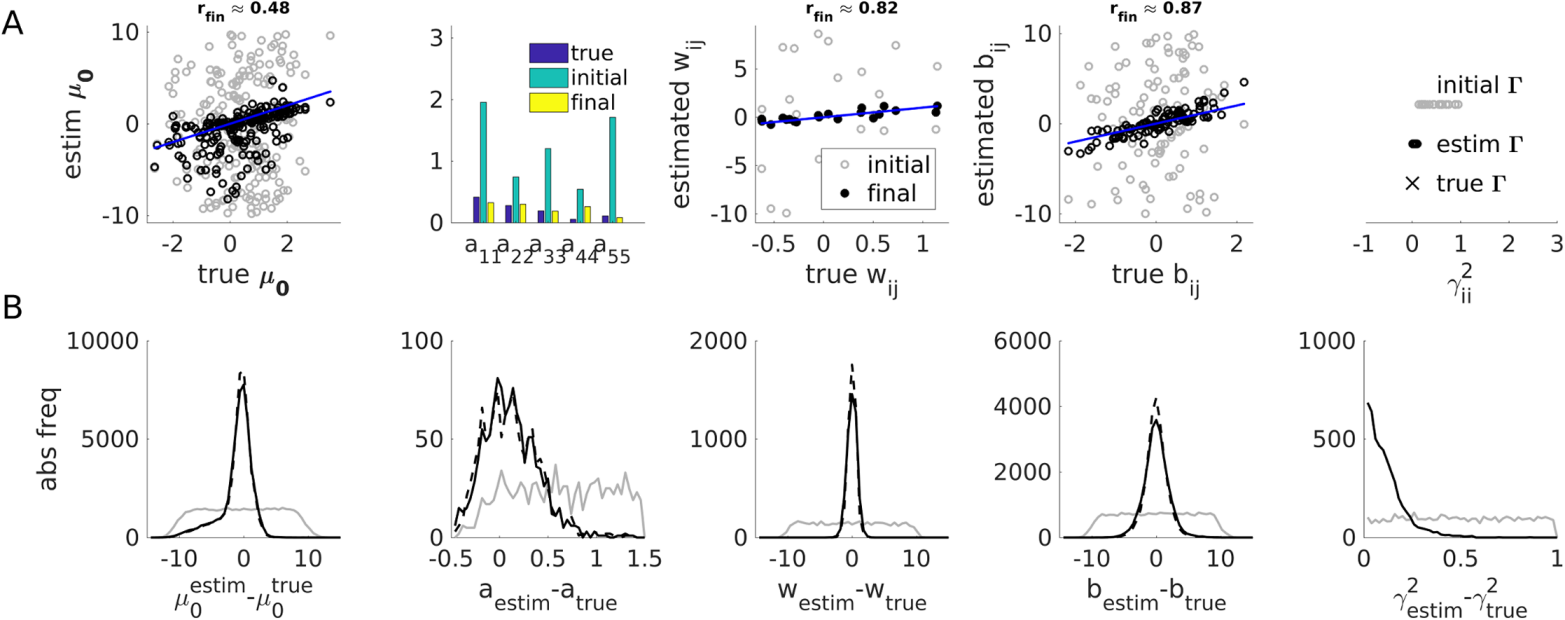
Full EM algorithm on working memory model. (A) Parameter estimates for ML solution from Fig 5. True parameters (on *x*-axes or as blue bars, respectively), initial (gray circles or green bars) and final (black circles or yellow bars) parameter estimates for (from left to right) **μ**_0_,**A**,**W**,**B**,**Γ**. Bisectrix lines in blue. Correlations between true and final estimates are indicated on top (note from Eq 17C that the estimates for **μ**_0_ are based on just one state, hence will naturally be less precise). (B) Distributions of initial (gray curves), final (black-solid curves), and final after reordering of states (black-dashed curves), deviations between estimated and true parameters across all 240 EM runs from different initial conditions. All final distributions were approximately centered around 0, indicating that final parameter estimates were relatively unbiased. Note that partial information about state assignments was implicitly provided to the network through the unit-specific inputs (and, more generally, may also come from the unit-specific thresholds *θ*_*i*_, although these were all set to 0 for the present example), and hence state reordering only produced slight improvements in the parameter estimates.

Fig 7 illustrates the distribution of initial and final parameter estimates around their true values across all 240 runs (before and after reordering the estimated latent states based on the rotation that would be required for achieving the optimal mapping onto the true states, as determined through Procrustes analysis). Fig 7 reveals that a) the EM algorithm does clearly improve the estimates and b) these final estimates seemed to be relatively ‘unbiased’ (i.e., with deviations centered around 0).

### Computational complexity of state inference and EM algorithm

**Fig 8:**
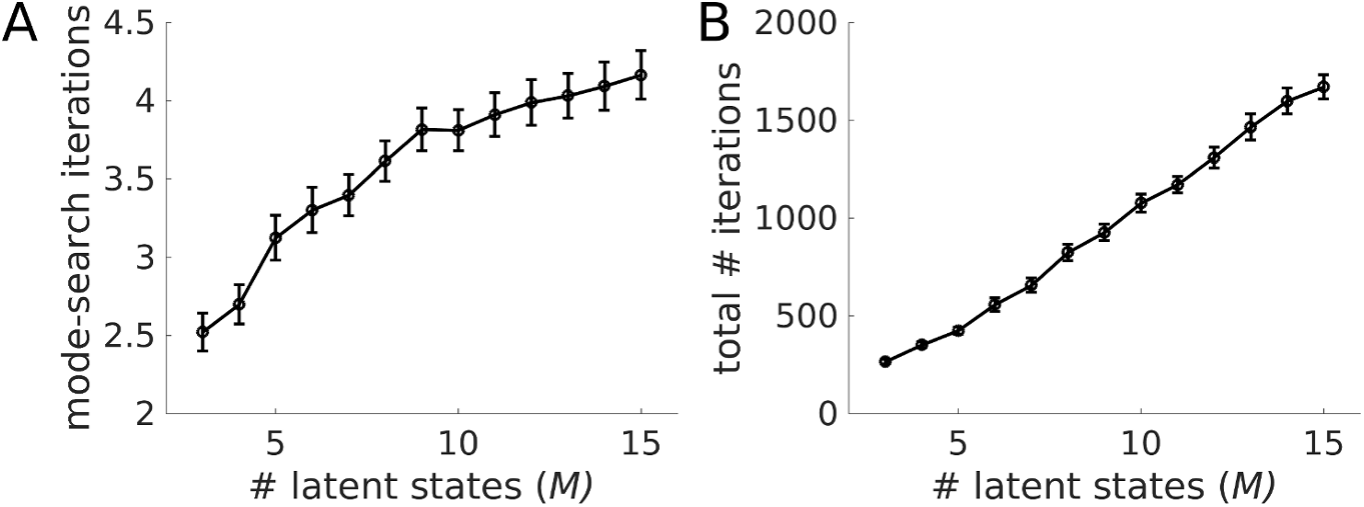
Computational performance of state inference (E-step) and full EM algorithm as the number of latent states is increased. (A) The number of full mode-search iterations, i.e. the number of constraint-sets **Ω** visited as defined through constraint vector **d** (cf. Eq 7) within one E-step, increases (sub-)linearly with the number *M* of latent states included in the model. (B) Likewise, the *total* number of mode-search steps (evaluated with *single-constraint* flipping here) summed across all EM iterations increases about linearly with *M* (single-constraint flipping requires about 10-fold more iterations than full-constraint flipping, but was observed to perform more stably). Note that this measure combines the number of EM iterations with the number of mode-search steps during each EM pass, and in this sense reflects the scaling of the full EM procedure. Performance tests shown were run on the experimental data sets illustrated in Figs 9–12. Means were obtained across 40 different initial conditions (with each, in turn, representing the mean from 3x14 = 42 runs in A, or 14 runs in B, separately for each of 14 trials). Error bars = SEM (across initial conditions).

How do the computational costs of the algorithm grow as the number of latent variables in the model is increased? As pointed out in Paninski et al. [16], exploiting the block-tridiagonal nature of the covariance matrices, the numerical operations within one iteration of the state inference algorithm (i.e., solving $\partial Q_{\Omega }^{*}(Z)/\partial Z=0$, Eq 7) can be done in linear, *O(M×T)*, time, just like with the Kalman filter (due to the model’s Markov properties, full inversion of the Hessian is also not necessary to obtain the relevant moments of the posterior state distribution). This leaves open the question of how many more mode search iterations, i.e. linear equation solving (Eq 7) and constraint-flipping (vector **d**_Ω_) steps, are required as the number of latent variables (through either *M* or *T*) increases. The answer is provided in Fig 8A which is based on the experimental data set discussed below. Although a full computational complexity analysis is beyond the scope of this paper, at least for these example data (and similar to what has sometimes been reported for the somewhat related Simplex algorithm; [56]), the increase with *M* appears to be at most linear. Likewise, the *total* number of iterations within the full EM procedure, i.e. the number of mode-search steps summed across *all* EM iterations (thus reflecting the overall scaling of the full algorithm), was about linear (Fig 8B; in this case, single-constraint instead of complete flipping (see Methods) was used which, of course, increases the overall number of iterations but may perform more stably; note that in general the absolute number of iterations will also depend on detailed parameter settings of the algorithm, like the EM convergence criterion and error tolerance). Thus, overall, the present state inference algorithm seems to behave quite favorably, with an at most linear increase in the number of iterations required as the number of latent variables is ramped up.

**Fig 9:**
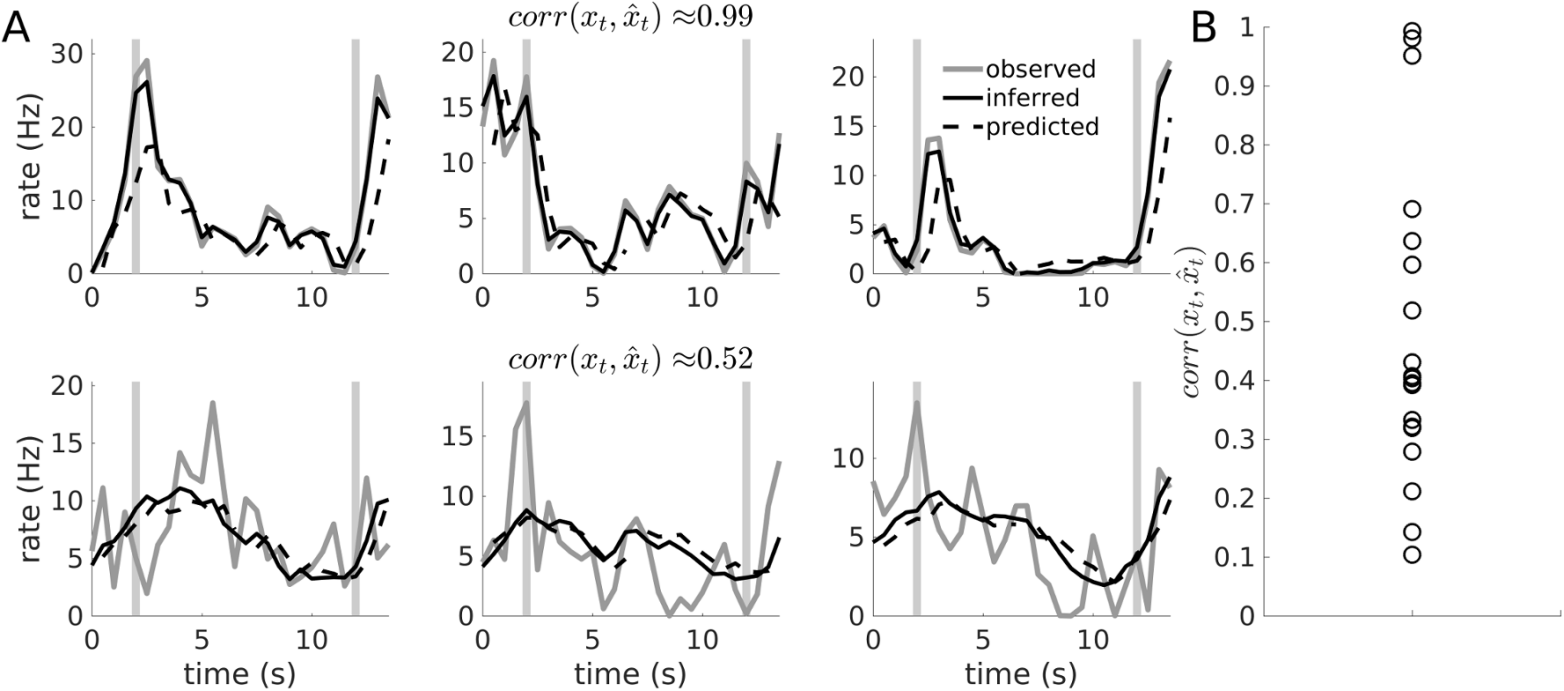
Prediction of single unit responses. (A) Top row: Example of an ACC unit (darker-gray curves) captured very well by the estimated PLRNN despite considerable trial to trial fluctuations (3 consecutive trials shown). Both model estimates from the directly inferred states (black curves) and from 1-step-ahead predictions of states **z**_t_ (dashed curves) are shown. Bottom row: Example of another ACC unit on the same three trials where only the average trend was captured by the PLRNN when firing rates were estimated from either the directly inferred or predicted states. Gray vertical bars in all panels indicate times of cue/ response. State estimation in this case was performed by inverting only the single constraint corresponding to the largest deviation on each iteration (see Methods). (B) Correlations among actual ($\left\{x_{t}\right\}$) and predicted ($\left\{{\overset{⌢}{x}}_{t}\right\}$) observations for all 19 neurons within this data set.

**Fig 10:**
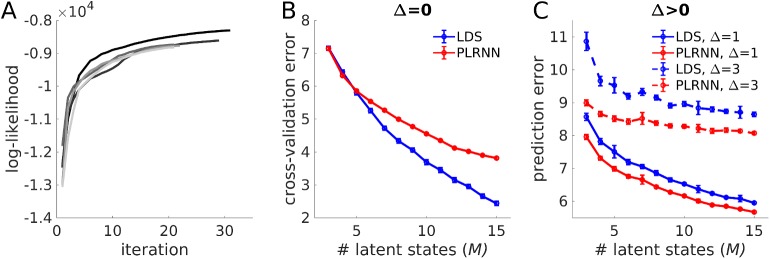
Log-likelihood of PLRNN and cross-validation performance of linear (LDS) and nonlinear (PLRNN) state space models on the ACC data. (A) Examples of log-likelihood curves across EM iterations from the 5/36 highest-likelihood runs for a 5-state PLRNN estimated from 19 simultaneously recorded prefrontal neurons on a working memory task (cf. Fig 9). State estimation here was performed by inverting only the single constraint corresponding to the largest deviation on each iteration (see Methods). (B) Cross-validation error (CVE) for the PLRNN (red curve) and the LDS (blue curve) as a function of the number of latent states *M*. CVE was assessed on each of 14 left-out trials with model parameters estimated from the remaining 14–1 = 13 experimental trials. Shown are squared errors ${\left(x_{it}-{\hat{x}}_{it}\right)}^{2}$ averaged across all units *i*, time points *t*, and 40 different initial conditions. (C) Same as A, but with outputs ${\hat{x}}_{it}$ estimated from states predicted Δ*t* = 1 (solid curves) or Δ*t* = 3 (dashed curves) time steps ahead. Note that in this case the PLRNN consistently performs better than a LDS for all *M*, with the PLRNN-LDS difference growing as Δ*t* increases. Error bars represent SEMs across those of the 40 initial conditions for which stable models were obtained (same for the means).

**Fig 11:**
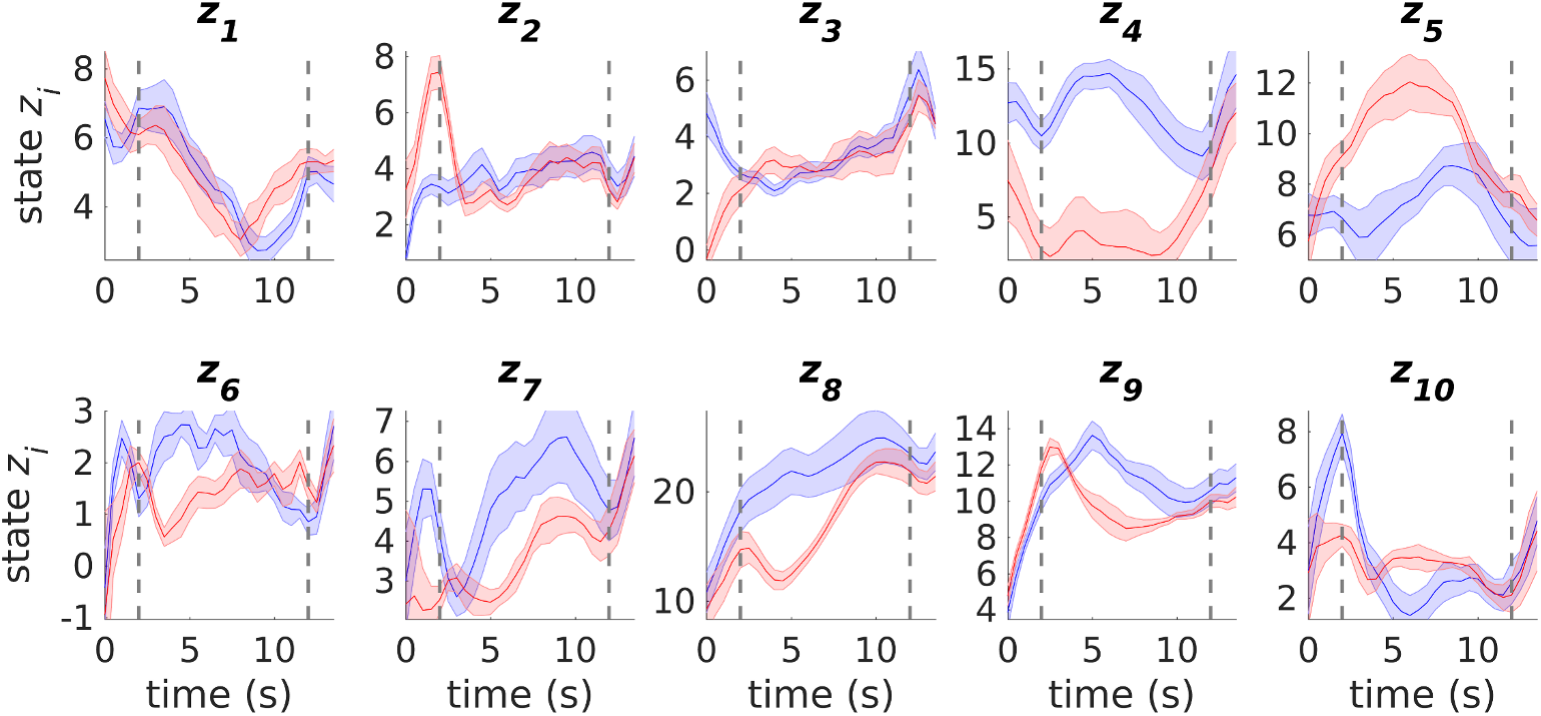
Example (from one of the 5 highest likelihood solutions) for latent states of a PLRNN with *M* = 10 estimated from ACC multiple single-unit recordings during working memory (cf. Figs 9 and 10). Shown are trial averages for left-lever (blue) and right-lever (red) trials with SEM-bands computed across trials. Dashed vertical lines flank the 10 s period of the delay phase used for model estimation. Note that latent variables *z*_4_ and *z*_5_, in particular, differentiate between left and right lever responses throughout most of the delay period.

**Fig 12:**
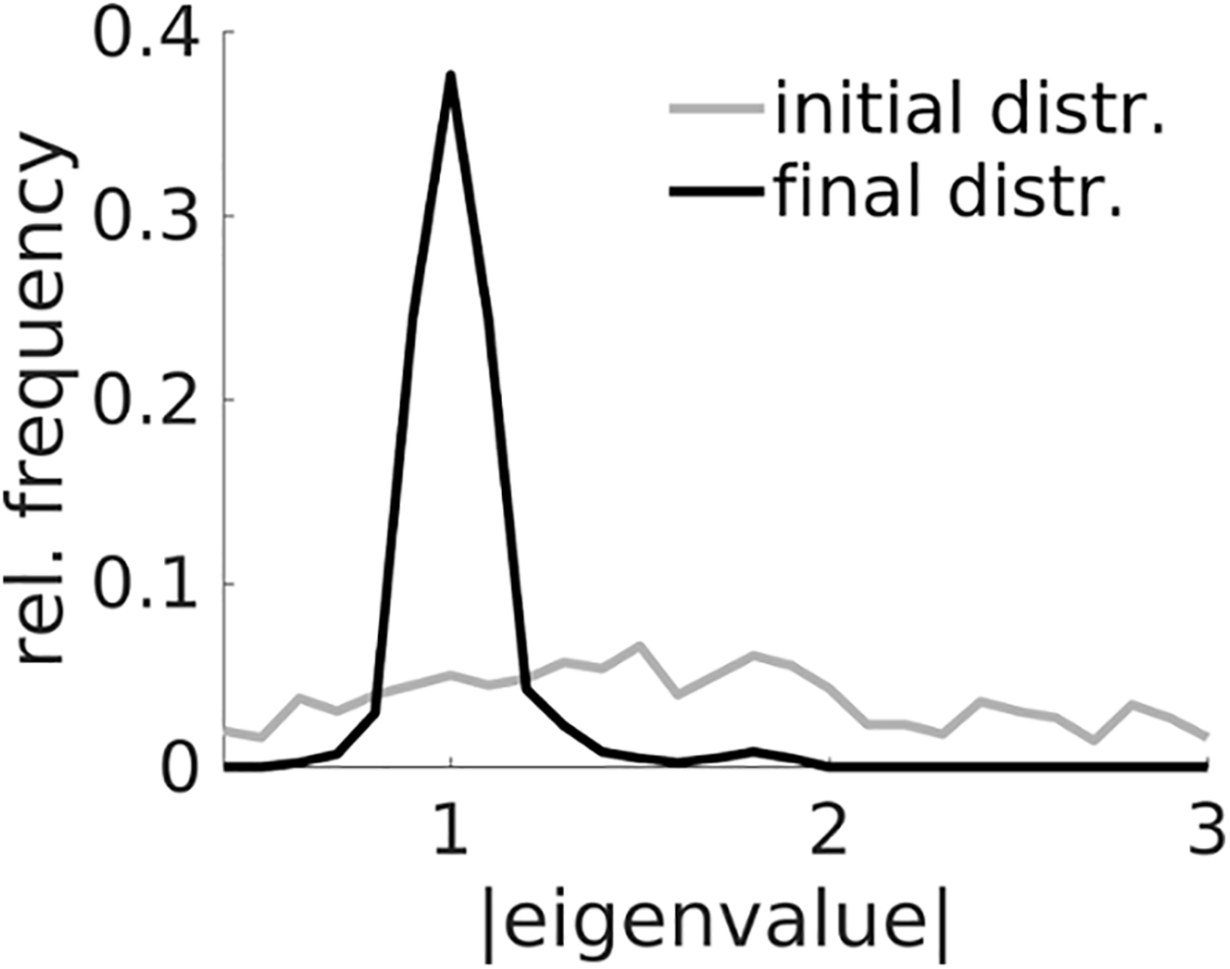
Initial (gray) and final (black) distributions of maximum (absolute) eigenvalues associated with all fixed points of 400 PLRNNs estimated from the experimental data (cf. Figs 9–11) with different initializations of parameters, including the (fixed) threshold parameters *θ*_*i*_. Initial parameter configurations were deliberately chosen to yield a rather uniform distribution of absolute eigenvalues ≤ 3.

### Application to experimental recordings

I next was interested in what kind of structure the present PLRNN approach would retrieve from experimental multiple (*N* = 19) single-unit recordings obtained while rats were performing a simple and well-examined working memory task, namely spatial delayed alternation [41] (see Methods). (Note that in the present context this analysis is mainly meant as an exemplification of the current model approach, not as a detailed examination of the working memory issue itself.) The delay was always initiated by a nose poke of the animal into a port located on the side opposite from the response levers, and had a minimum length of 10 s. Spike trains were first transformed into kernel density estimates by convolution with a Gaussian kernel (see Methods), as done previously (e.g. [12; 57; 58]), and binned with 500 ms resolution. This also renders the observed data more suitable to the Gaussian noise assumptions of the present observation model, Eq 3. Models with different numbers of latent states were estimated, with *M* = 5 or *M* = 10 chosen for the examples below. Periods of cue presentation were indicated to the model by setting external inputs *s*_*it*_ = 1 to units *i* = 1 (left lever) or *i* = 2 (right lever) for three 500 ms time bins surrounding the event (and *s*_*it*_ = 0 otherwise), and the response period was indicated by setting *s*_3*t*_ = 1 for 3 consecutive time bins irrespective of the correct response side (i.e., non-discriminatively). The EM algorithm was started from a range of different initializations of the parameters (including thresholds **θ**), and the 5 highest likelihood solutions were considered further for the examples below.

Fig 10A gives the log-likelihoods across EM iterations for these 5 highest-likelihood solutions (starting from 36 different initializations) for the *M* = 5 model. Interestingly, there were single neurons whose responses were predicted quite well by the estimated model despite large trial-to-trial fluctuations (Fig 9A, top row), while there were others with similar trial-to-trial fluctuations for which the model only captured the general trend (Fig 9A, bottom row; to put this into context, Fig 9B gives the full distribution of correlations between actual and predicted observations across all 19 neurons). This may potentially indicate that trial-to-trial fluctuations in single neurons could be for very different reasons: For instance, in those cases where strongly varying single unit responses are nevertheless tightly reproduced by the estimated model, a larger proportion of their trial-to-trial fluctuations may have been captured by the latent state dynamics, ultimately rooted in different (trial-unique) initializations of the states (recall that the states are not completely free to vary in accounting for the observations, but are constrained by the model’s temporal consistency requirements). In contrast, when only the average trend is captured, the neuron’s trial-to-trial fluctuations may be more likely to represent true intrinsic (or measurement) noise sources that the model’s deterministic part cannot account for. In practice, such conclusions would have to be examined more carefully to rule out that no other factors in the estimation procedure, like different local maxima, initializations, or over-fitting issues (see below), could account for these differences. Although this was not further investigated here, this observation nevertheless highlights the potential of (nonlinear) state space models to possibly provide new insights also into other long-standing issues in neurophysiology.

Cross-validation is an established means to address over-fitting [45], although due to the presence of both unknown parameters and unknown states, its application to state space models and its interpretation in this context may be a bit less straightforward. Here the cross-validation error was first assessed by leaving out each of the 14 experimental trials in turn, estimating model parameters **Ξ** from the remaining 13 trials, inferring states **z**_*t*_ given these parameters on the left-out trial, and computing the squared prediction errors ${\left(x_{it}-{\hat{x}}_{it}\right)}^{2}$ between actual neural observations *x*_*it*_ and model predictions ${\hat{x}}_{it}$ on the left-out trial. As shown in Fig 10B, this measure steadily (albeit sub-linearly) decreases as the number *M* of latent states in the model is increased. At first sight, this seems to suggest that with *M* = 5 or even *M* = 10 the over-fitting regime is not yet reached. On the other hand, the latent states are, of course, not completely fixed by the transition equations, but have some freedom to vary as well (the true effective degrees of freedom for such systems are in fact very hard to determine, cf. [59]). Hence, we also examined the Δ*t*-step-ahead prediction errors, that is, when the transition model were iterated Δ*t* steps forward in time, and ${\hat{x}}_{i,t+\Delta t}=b_{i\cdot }\phi ({\hat{z}}_{t+\Delta t})$ estimated from the deterministically *predicted* states ${\hat{z}}_{t+\Delta t}=H^{\Delta t}(E[z_{t}])$ (with H^Δ*t*^ the Δ*t*-times iterated map H(**z**_*t*_) = **Az**_*t*_ + **W***ϕ*(**z**_*t*_) + **Cs**_*t*_), not from the directly inferred states (that is, predictions were made on data points which were neither used to estimate parameters nor to infer the current state). These curves are shown for Δ*t* = 1 and Δ*t* = 3 in Fig 10C, and confirm that *M* = 5 might be a reasonable choice at which over-fitting has not yet ensued. (Alternatively, the predictive log-likelihood, $\log\;p(X^{test}|{\hat{\Xi }}^{train})=\log\int p(X^{test}|\hat{Z})p(\hat{Z}|{\hat{\Xi }}^{train})d\hat{Z}$, may be used for model selection (i.e., choice of *M*), with $p(\hat{Z}|{\hat{\Xi }}^{train})$ either approximated through the E-step algorithm (with all **X**-dependent terms removed), or bootstrapped by generating $\hat{Z}$-trajectories from the model with parameters ${\hat{\Xi }}^{train}$ (note that this is different from particle filtering since $p(\hat{Z}|{\hat{\Xi }}^{train})$ does not depend on test observations **X**^*test*^). This is of course, however, computationally more costly to evaluate than the Δ*t*-step-ahead prediction error.)

Fig 11 shows trial-averaged latent states for both left- and right-lever trials, illustrated in this case for one of the five highest likelihood solutions (starting from 100 different initializations) for the *M* = 10 model. Recall that the first 3 PLRNN units received external inputs to indicate left cue (*i* = 1), right cue (*i* = 2), or response (*i* = 3) periods, and so, not too surprisingly, reflect these features in their activation. On the other hand, the cue response is not very prominent in unit *i* = 1, indicating that activity in the driven units is not completely dominated by the external regressors either, while unit *i* = 10 (not externally driven) shows a clear left-cue response. Most importantly, many of the remaining state variables clearly distinguish between the left and right lever options throughout the delay period of the task, in this sense carrying a memory of the cue (previous response) within the delay. Some of the activation profiles appear to systematically climb or decay across the delay period, as reported previously (e.g. [1; 60]), but are a bit harder to read (at least in the absence of more detailed behavioral information), such that one may want to stick with the simpler *M* = 5 model discussed above. Either way, for this particular data set, the extracted latent states appear to summarize quite well the most salient computational features of this simple working memory task.

Further insight about the dynamical mechanisms of working memory might be gained by examining the system’s fixed points and their eigenvalue spectrum. For this purpose, the EM algorithm was started from 400 different initial conditions (that is, initial parameter estimates and threshold settings **θ**) with maximum absolute eigenvalues (of the corresponding fixed points) drawn from a relatively uniform distribution within the interval [0 3]. Although the estimation process rarely returned truly multi-stable solutions (just 2.5% of all cases), one frequently discussed candidate mechanism for working memory (e.g. [29; 32]), there was a clear trend for the final maximum absolute eigenvalues to aggregate around 1 (Fig 12). For the discrete-time dynamical system (1) this implies it is close to a bifurcation, with fixed points on the brink of becoming unstable, and will tend to produce (very) slow dynamics as the degree of convergence shrinks to zero along the maximum eigenvalue direction (strictly, a single eigenvalue near 1 does not yet guarantee a slow approach, but makes it very likely, especially in a (piecewise) linear system). Indeed, effectively slow dynamics is all that is needed to bridge the delays (see also [1]), while true multi-stability may perhaps even be the physiologically less likely scenario (e.g. [61; 62]). (Reducing the bin width from 500 ms to 100 ms appeared to produce solutions with eigenvalues even closer to 1 while retaining stimulus selectivity across the delay, but this observation was not followed up more systematically here).

### Comparison to linear dynamical systems

Linear dynamical systems (LDS) have frequently and successfully been used to infer smooth neural trajectories from spike train recordings [15; 16; 20; 22] or other measurement modalities [63]. However, as noted before, they cannot, on their own, as a matter of principle, produce a variety of dynamical phenomena essential for neural computation and observed experimentally, including multi-stability (e.g. [29; 2]), limit cycles (*stable* oscillations; e.g. [3]), chaos (e.g. [33]), and many types of bifurcations and phase transitions. For instance, the question of whether working memory performance is better explained in terms of multi-stability or effectively slow dynamics (see above, Fig 12) is largely beyond the realm of an LDS, due to its inherent inability to express multi-stability in the first place. An LDS is therefore less suitable for retrieving system dynamics or computations in general.

Nevertheless, it may still be instructive to ask how much of the underlying dynamics could already be explained in linear terms. The most direct comparison of PLRNN to LDS performance is made by replacing the nonlinearity *ϕ*(**z**_*t*_) = max{**0**,**z**_*t*_ − **θ**} in Eq 1 simply by the linear function *ϕ*(**z**_*t*_) = **z**_*t*_−**θ**, yielding an LDS with exactly the same parameters **Ξ** as the PLRNN which can be subjected to the very same estimation and inference procedures (only that state inference can now be done exactly in just one step). Fig 10B reveals that a LDS fits the observed neural recordings about as well as the PLRNN for *M*≤5, and starts to excel PLRNN performance for *M*>5. Since the major difference in this context is that the PLRNN places a tighter constraint on the temporal consistency of the states through the threshold-nonlinearity, it seems reasonable that this result is due to over-fitting, i.e. the LDS due to its smoothness allows for more freedom for the states to adjust to the actual observations (cf. [64]). It is important to bear in mind that consistency with the actual observations is just one objective of the maximum-likelihood formulation, Eq 5; the other is consistency of states across time according to the model specification.

Either way, the PLRNN starts to significantly outperform the LDS in terms of the Δ*t*-step-ahead prediction errors (see above), with the gap in performance widening as Δ*t* increases (Fig 10C). This strongly suggests that the PLRNN has internalized aspects of the system dynamics which the LDS fails to represent, i.e. supports the presence of nonlinear structure in the transition dynamics. Interestingly, looking back at Fig 5B, it turns out that even for simulated data generated by a PLRNN (at least for this example), for Δ*t* = 0 an estimated LDS is about as good in reproducing the actual observations as an estimated PLRNN itself (with an MSE close to 0), that is, although, unlike the PLRNN it does not have the correct model structure. However, similar to what has been observed for the experimental data (Fig 10B and 10C), this performance rapidly drops and falls far behind that of the PLRNN (which remains low) as 1 or more time steps into the future are to be predicted (note that for the simulated model, unlike the experimental example, the true number of states is known of course). This confirms that although the LDS may capture the actual observations quite well, it may not, unlike the PLRNN, be able to properly represent the underlying system within its internal dynamics.

As a note on the side, an LDS could be utilized to find proper, efficient initializations for the corresponding PLRNN, or to first improve initial estimates (although it remains to be examined whether this could potentially also bias the search space in an unfavorable way).

## Discussion

### Reconstructing computational dynamics from neuronal recordings

In the present work, a semi-analytical, maximum-likelihood (ML) approach for estimating piecewise-linear recurrent neural networks (PLRNN) from brain recordings was developed. The idea is that such models would provide 1) a representation of neural trajectories and computationally relevant dynamical features underlying high-dimensional experimental time series in a much lower-dimensional latent variable space (cf. [20; 25]) and 2) more direct access to the neural system’s statistical and computational properties. Specifically, once estimated to reproduce the data (in the ML sense), such models may, in principle, allow for more detailed analysis and in depth insight into the system’s probabilistic computational dynamics, e.g. through an analysis of fixed points and their linear stability (e.g. [28; 30; 32; 47; 65–70]), properties which are not directly accessible from the experimental time series.

Model-free (non-parametric) techniques, usually based on Takens’ delay embedding theorem [71] and extensions thereof [72; 73], have also frequently been applied to gain insight into neuronal dynamics and its essential features, like attracting states associated with different task phases from in-vivo multiple single-unit recordings [11; 12] or unstable periodic orbits extracted from relatively low-noise slice recordings [74]. In neuroscience, however, one commonly deals with high-dimensional observations, as provided by current multiple single-unit or neuroimaging techniques (which still usually constitute just a minor subset of all the system’s dynamical variables). In addition, there is a large variety of both process and measurement noise sources. Measurement noise may come from direct physical sources like, for instance, instabilities and movement in the tissue surrounding the recording electrodes, noise properties of the recording devices themselves, the mere fact that only a fraction of all system variables is experimentally accessed (‘sampling noise’), or may result from preprocessing steps like spike sorting (e.g. [75; 76]). Process noise sources include thermal fluctuations and the probabilistic behavior of single ion channel gating [77], probabilistic synaptic release [6], fluctuations in neuromodulatory background and hormone levels, and a large variety of uncontrollable external noise sources via the sensory surfaces, including somatosensory and visceral feedback from within the body. In fact, the stochasticity of the neural dynamics itself has been deemed essential for a number of computational processes like those involved in decision making and inference [7–9]. This is therefore a quite different scenario from the comparatively low-dimensional and low-noise situations in, e.g., laser physics [78], and delay-embedding-based approaches to the reconstruction of neural dynamics may have to be augmented by machine learning techniques to retrieve at least some of its most salient features [11; 12].

Of course, model-based approaches like the one developed here are also plagued by the high dimensionality and high noise levels inherent in neural data, but perhaps to a lesser extent than approaches like delay embeddings that aim to directly construct the state space from the observations (see also [79]). This is because models as pursued in the statistical state space framework explicitly incorporate process and measurement noise assumptions into the system’s description, performing smoothing in the latent space. Also, as long as the latent variable space itself is relatively small and related to the observations by simple linear equations, as here, the high dimensionality of the observations themselves does not constitute a too serious issue for estimation. More importantly, however, it is of clear advantage to have access to process equations generating state distributions consistent with the observations, as this allows for a more in depth analysis of the system’s stochastic dynamics and its relation to neural computation (e.g. [2; 28; 30; 47; 68; 70; 33]). There have also been various attempts to account for the observed dynamics directly in terms of nonlinear time series models (e.g. [13, 78, 80]), i.e. without reference to an underlying latent variable model, e.g. through differential equations expressed in terms of nonlinear basis expansions in the observations, estimated through strongly regularized (penalized) regression methods [11; 13; 80]. For neuroscientific data where usually only a small subset of all dimensions is observed, this implies that this approach has to be augmented by delay embedding techniques to replace the unobserved variables. This, in turn, may potentially lead to very high-dimensional systems (cf. [11,13]) that may necessitate further pre-processing steps to reduce the dimensionality again, in a way that preserves the dynamics. Also, there is no distinction between measurement and dynamical noise in these models, and, although functionally generic, the parameters of such models may be harder to interpret in a neuroscientific context. How these different assumptions and methodological steps affect the reconstruction of neural dynamics from high-dimensional, noisy neural time series, as compared to state space models, remains an open and interesting question at this point.

### Comparison to other neural state space models

State space models are a popular statistical tool in many fields of science (e.g. [14; 63]), although their applications in neuroscience are of more recent origin [15, 16; 18; 19; 21–24]. The Dynamic Causal Modeling (DCM) framework advanced in the human fMRI literature to infer the functional connectivity of brain networks and their dependence on task conditions [63; 81] may be seen as a state space approach, although these models usually do not contain process noise (except for the more recently proposed ‘stochastic DCM’ [81]) and are commonly estimated through Bayesian inference, which imposes more constraints (via computational burden) on the complexity of the models that could potentially be dealt with in this framework. In neurophysiology, Smith & Brown [15] were among the first to suggest a state space model for multivariate spike count data by coupling a linear-Gaussian transition model with Poisson observations, with state estimation achieved by making locally Gaussian approximations to Eq 18. Similar models have variously been used subsequently to infer local circuit coding properties [18] or, e.g., biophysical parameters of neurons or synaptic inputs from postsynaptic voltage recordings [82; 17]. Yu et al. [25] proposed Gaussian Process Factor Analysis (GPFA) for retrieving lower-dimensional, smooth latent neural trajectories from multiple spike train recordings. In GPFA, the correlation structure among the latent variables is specified (parameterized) explicitly rather than being given through a transition model. Buesing et al. [20], finally, discuss regularized forms of neural state space models to enforce their stability.

By far most of the models discussed above are linear in their latent dynamics, however (although observations may be non-Gaussian). As demonstrated in the Results, linear state space models may potentially be similarly well fit for reproducing actual observations, at least for the particular model and experimental systems studied here. In fact, this is not at all guaranteed in general, if the underlying processes are highly nonlinear (unlike those in Fig 5 where the nonlinearity was comparatively mild (not depending on multi-stability)). Thus, they may often be sufficient to obtain smoothed neural trajectories or lower-dimensional representations of the observed process [25], to uncover properties of the underlying connectivity [63; 81], or to estimate synaptic/neuronal parameters [16; 82]. However, as linear systems are strongly limited in the repertoire of dynamics and computations they can produce (e.g. [65; 83]), they cannot serve as a model for the underlying computational processes and dynamics in general, and do not allow for the type of analyses which led into Fig 12. A LDS can, on its own, express at most one *isolated* fixed point (or a neutrally un-/stable continuum), or (neutrally un-/stable) sinusoidal-like cycles, but cannot represent any of the more complex phenomena which characterize physiological activity and are a hallmark of most computation. On the other hand, a direct comparison of LDS vs. PLRNN predictive performance may be highly revealing in itself: While some cognitive processes (like decision making, sequence or syntax generation) would clearly be expected to be highly nonlinear in their underlying dynamics [4; 84; 85], others (early stimulus responses, or value updating, for instance) may follow more of a linear rule (e.g., if stimuli were projected into a high-dimensional space for linear separability; cf. [86]). Directly contrasting LDS with PLRNN predictions on the same data set (as carried out in Fig 10), may uncover such important differences in computational mechanisms, and hence constitute an interesting analysis strategy in its own right.

There are a couple of other exceptions from the linear framework the current work builds on: Yu et al. [23] suggested a RNN with sigmoid-type activation function (using the error function), coupled to Poisson spike count outputs, and used it to reconstruct the latent neural dynamics underlying motor preparation and planning in non-human primates. In their work, they combined the Gaussian approximation suggested by Smith & Brown [15] with the Extended Kalman Filter (EKF) for estimation within the EM framework. These various approximations in conjunction with the iterative EKF estimation scheme may be quite prone to numerical instabilities and accumulating errors, however (cf. [26]). Earlier work by Roweis & Ghahramani [27] used radial basis function (RBF) networks as a partly analytically tracktable approach. Nonlinear extensions to DCM, incorporating quadratic terms, have been proposed as well recently [87]. State and parameter estimation has also been attempted in (noisy) nonlinear biophysical models [88; 89], but these approaches are usually computationally expensive, especially when based on numerical sampling [89], while at the same time pursuing objectives somewhat different from those targeted here (i.e., less focused on computational properties). A very recent article by Whiteway & Butts [90] discusses an approach closely related to the present one in that it also assumed piecewise linear latent states (or, ‘rectified linear units (ReLU)’). Unlike here, however, the latent states were not connected through a dynamical systems model with separate process noise (but just constrained through a smoothness prior). Indeed, the objectives of this work were different, as Whiteway & Butts [90] aimed more at capturing unobserved sources of input in accounting for observed neural activity (more in the spirit of factor analysis), rather than attempting to retrieve an underlying stochastic dynamics as in the present work. They found, however, that the inclusion of nonlinearities may help in accounting for observed data and improve interpretability of the latent factors.

In summary, nonlinear neural state space models remain a relatively under-researched topic in theoretical neuroscience. PLRNNs, as chosen here, have the advantage of being mathematically comparatively tracktable, which allowed for the present, reasonably fast, semi-analytical algorithm, yet they are computationally and dynamically still powerful [91–94].

### Alternative inference/training schemes, network architectures, and observation distributions

A number of other inference schemes have been suggested for state space models, comprising both analytical approximations [22] and numerical (sampling) techniques (e.g. [26]). Among the former are the Extended Kalman filter (based on local Taylor series approximations), methods based on variational inference as reviewed in Macke et al. [22], or the (global) Laplace approximation advertized in Paniniski et al. ([16]; see also [22]). Durbin & Koopman [26] review different variants of particle filter schemes for sequential numerical sampling. These may often be simpler to use, but are usually computationally much more costly than the semi-analytical methods. The Unscented Kalman Filter may be seen somewhere in between, using a few deliberately chosen sample (‘sigma’) points for a local parametric assessment [26]. Here we chose a global approach rather than a recursive-sequential scheme, that is by solving the full *M×T* system of linear equations within each subspace defined by constraints Ω in one go. Apart from its generally nice computational properties as discussed in Paniniski et al. [16], it seems particularly well-suited for the present piecewise-linear model Eqs (1) and (3), in dealing with the combinatorial explosion which builds up along the chain from *t* = 1…*T*. However, the mathematical properties of the present algorithm, among them issues of convergence/monotonicity, local maxima/ saddles, and uniqueness and existence of solutions, certainly require further illumination which may lead to algorithmic improvements. In particular, identifiability of dynamics, that is to what degree and under which conditions the true underlying dynamical system could be recovered by the PLRNN-EM approach, remains an open issue (one line of extension toward greater approximation power would be polynomial basis expansions, at the cost, however, of losing the straightforward interpretation in terms of ‘neural networks’).

Most commonly, different variants of gradient-based techniques are being used to train recurrent neural networks to fit observations [40, 42, 50, 95, 96]. For instance, recurrent network models have been trained to perform behavioral tasks [43] or reproduce behavioral data to infer the dynamical mechanisms potentially underlying working memory [97] or context-dependent decision making [68]. In these settings, however, the observations–that is behavioral data points or requested task outputs–are usually relatively sparse in time compared to the time scale of the underlying dynamics, unlike the neural time series settings studied here where the data can be as dense as the latent state vectors of the model. More importantly, in contrast to these previous gradient-based approaches, the present scheme embeds RNNs into a statistical framework that comes with explicit probability assumptions, thereby puts error bars on state and parameter estimates and returns the posterior probability distribution across latent states, which links in with the observations through a separate measurement function (enabling, for instance, dimensionality reduction), and allows for likelihood-based statistical inference and model comparison. Some preliminary analyses using stochastic Adagrad [98] for training PLRNNs on the time series from the working memory example (cf. Fig 3) seemed, on top, to indicate that the resulting parameter estimates may correlate less well (<0.51 for **A** and **W**, after optimal reordering of states) with the true model parameters than those obtained with the present EM approach (>0.78) for the lowest error/ highest likelihood solutions (this may potentially be improved through teacher forcing, which, however, is not applicable when the observed and latent space differ in dimensionality and are related through an, in general, not strictly invertible transform, as here).

Other observation models, like the Poisson model for spike counts [15; 22], are also relatively straightforward to accommodate within this framework (see [16]). However, there are also other ways to deal with spike count observations, like simple Box-Cox-type transforms to make them more Gaussian, e.g. the sqrt-transform suggested for GPFA [25], or kernel-density smoothing (e.g. [58]) as used here. The latter has the additional advantage of reducing the impact of ‘binning noise’, due to the somewhat arbitrary mapping of real-valued spike times onto discrete (user-defined) time bins for the purpose of counting. In general, from a practical perspective, it may therefore still be an open question of whether the additional computational burden that comes with non-Gaussian observation models (e.g. the requirement of Newton-Raphson steps for each mode-search iteration) pays off in the end compared to these alternatives. In either case, for the time being, it seems useful to have a more general approach which can also deal with other measurement modalities, like neuroimaging data, which are not of a count-nature.

The present approach could also be extended by incorporating various additional structural features. For instance, a distinction between units with excitatory vs. inhibitory connections [43] could be accommodated quite easily within the present framework (requiring constrained optimization for weight parameters, however, e.g. through quadratic programming). Or special gated linear units which make LSTM networks so powerful [39,40] may potentially also yield improvements within the present EM/ state-space framework (although, in general, one may want to be cautious about the assumptions that additional structural elements like these may imply about the underlying neural system to be examined).

### Mechanisms of working memory

Although the primary focus of this work was to develop and evaluate a state space framework for PLRNNs, some discussion of the applicational example chosen here, working memory, is in order. Working memory is generally defined as the ability to actively hold an item in memory, in the absence of guiding external input, for short-term reference in subsequent choice situations [99]. Various neural mechanisms have been proposed to underlie this cognitive capacity, most prominently multi-stable neural networks which retain short-term memory items by switching into one of several stimulus-selective attractor states [28; 29; 32]. These attractors usually represent fixed points in the firing rates, with assemblies of recurrently coupled stimulus-selective cells exhibiting high rates while those cells not coding for the present stimulus in short-term memory remaining at a spontaneous low-rate base level. These models were inspired by the physiological observation of ‘delay-active’ cells [100–102], that is cells that switch into a high-rate state during the delay periods of working memory tasks, and back to a low-rate state after completion of a trial, similar to the ‘delay-active’ latent states observed in Fig 11. Nakahara & Doya [103] were among the first to point out, however, that, for working memory, it may be completely sufficient (or even advantageous) to tune the system close to a bifurcation point where the dynamics becomes very slow (see also [1]), and true multi-stability may not be required. This is supported by the present observation that most of the estimated PLRNN models had fixed points with eigenvalues close to 1 but were not truly bi- or multi-stable (cf. Fig 12), yet this was sufficient to account for maintenance of stimulus-selectivity throughout the 10 s delay of the present task (cf. Fig 11) and for experimental observations (cf. Fig 9). Recently, a number of other mechanisms for supporting working memory, however, including sequential activation of cell populations [104] or synaptic mechanisms [105] have been discussed. Thus, the neural mechanisms of working memory remain an active research area to which statistical model estimation approaches as developed here may contribute, but too broad a topic in its own right to be covered in more depth by this mainly methodological work.

## Models and methods

### Expectation-maximization algorithm: State estimation

As with most previous work on estimation in (neural) state space models [20; 22; 23; 26], we use the Expectation-Maximization (EM) framework for obtaining estimates of both the model parameters and the underlying latent state path. Due to the piecewise-linear nature of model (1), however, the conditional latent state path density p(**Z**|**X**) is a high-dimensional ‘mixture’ of partial Gaussians, with the number of integrations to perform to obtain moments of p(**Z**|**X**) scaling as 2^*T*×*M*^. Although analytically accessible, this will be computationally prohibitive for almost all cases of interest. Our approach therefore focuses on a computationally reasonably efficient way of searching for the mode (maximum a-posteriori, MAP estimate) of p(**Z**|**X**) which was found to be in good agreement with E(**Z**|**X**) in most cases. Covariances were then approximated locally around the MAP estimate.

More specifically, the EM algorithm maximizes the expected log-likelihood of the joint distribution p(**X**,**Z**) as a lower bound on log *p*(**X**|**Ξ**) [27], where **Ξ** = {**μ**_0_,**A**,**W**,**Σ**,**B**,**Γ**} denotes the set of to-be-optimized-for parameters (note that we dropped the thresholds **θ** from this for now):

$$\begin{array}{l} Q(\Xi ,Z)≔E[\log\;p(Z,X|\Xi )]\\ \;=E\left[-\frac{1}{2}{\left(z_{1}-\mu_{0}-s_{1}\right)}^{T}\Sigma^{-1}(z_{1}-\mu_{0}-s_{1})\right]\\ \;+E\left[-\frac{1}{2}\sum_{t=2}^{T}{\left(z_{t}-Az_{t-1}-W\phi (z_{t-1})-s_{t}\right)}^{T}\Sigma^{-1}(z_{t}-Az_{t-1}-W\phi (z_{t-1})-s_{t})\right]\\ \;+E\left[-\frac{1}{2}\sum_{t=1}^{T}{\left(x_{t}-B\phi (z_{t})\right)}^{T}\Gamma^{-1}(x_{t}-B\phi (z_{t}))\right]-\frac{T}{2}(\log|\Sigma |+\log|\Gamma |). \end{array}$$ (5)

For state estimation (*E*-step), if *ϕ* were a linear function, obtaining *E*(**Z**|**X**,**Ξ**) would be equivalent to maximizing the argument of the expectancy in (5) w.r.t. **Z**, i.e., E[**Z**|**X**,**Ξ**] ≡ arg max_Z_[log *p*(**Z**,**X**|**Ξ**)] (see [16]; see also [106]). This is because for a Gaussian mean and mode coincide. In our case, p(**X**,**Z**) is piecewise Gaussian, and we still take the approach (suggested in [16]) of maximizing log *p*(**Z**,**X**|**Ξ**) directly w.r.t. **Z** (essentially a Laplace approximation of *p*(**X**|**Ξ**) where we neglect the Hessian which is constant around the maximizer; cf. [16, 48]).

Let Ω(*t*) ⊆ {1…*M*} be the set of all indices of the units for which we have *z*_*mt*_ ≤ *θ*_*m*_ at time *t*, and **W**_Ω(*t*)_ and **B**_Ω(*t*)_ the matrices **W** and **B**, respectively, with all columns with indices ∈ Ω(*t*) set to 0. The state estimation problem can then be formulated as

$$\begin{array}{l} \;maximize\\ Q_{\Omega }^{*}(Z)≔-\frac{1}{2}{\left(z_{1}-\mu_{0}-s_{1}\right)}^{T}\Sigma^{-1}(z_{1}-\mu_{0}-s_{1})\\ \;-\frac{1}{2}\sum_{t=2}^{T}{\left[z_{t}-(A+W_{\Omega (t-1)})z_{t-1}+W_{\Omega (t-1)}\theta -s_{t}\right]}^{T}\Sigma^{-1}[z_{t}-(A+W_{\Omega (t-1)})z_{t-1}+W_{\Omega (t-1)}\theta -s_{t}]\\ \;-\frac{1}{2}\sum_{t=1}^{T}{\left(x_{t}-B_{\Omega (t)}z_{t}+B_{\Omega (t)}\theta \right)}^{T}\Gamma^{-1}(x_{t}-B_{\Omega (t)}z_{t}+B_{\Omega (t)}\theta )\\ w.r.t.\;(\Omega ,Z),\;subject\;to\;z_{mt}\leq \theta_{m}\;\forall t,m\in \Omega (t)\;\text{AND}\;z_{mt}>\theta_{m}\;\forall t,m\notin \Omega (t) \end{array}$$ (6)

Let us concatenate all state variables into one long column vector, **z** = (**z**_1_,…,**z**_*T*_) = (*z*_11_…*z*_*mt*_…*z*_*MT*_)^*T*^, and unwrap the sums across time into large, block-banded *MT×MT* matrices (see [16; 83]) in which we combine all terms quadratic or linear in **z**, or *ϕ*(**z**), respectively. Further, define **d**_Ω_ as the binary (*MT×*1) indicator vector which has 1s everywhere except for the entries with indices ∈ **Ω** ⊆ {1…*MT*} which are set to 0, and let **D**_Ω_ ≔ *diag*(**d**_Ω_) the *MT×MT* diagonal matrix formed from **d**_Ω_. Let **Θ** ≔ (**θ**,**θ**,…,**θ**)_(*MT*×1)_, and **Θ**^+*M*^ the same vector shifted downward by *M* positions, with the first *M* entries set to 0. One may then rewrite $Q_{\Omega }^{*}(Z)$ in the form

$$\begin{array}{l} Q_{\Omega }^{*}(Z)=-\frac{1}{2}[z^{T}(U_{0}+D_{\Omega }U_{1}+U_{1}^{T}D_{\Omega }+D_{\Omega }U_{2}D_{\Omega })z\\ \;-z^{T}(v_{0}+D_{\Omega }v_{1}+V_{2}diag[d_{\Omega }^{+M}]\Theta^{+M}+V_{3}D_{\Omega }\Theta +D_{\Omega }V_{4}D_{\Omega }\Theta )\\ \;-{\left(v_{0}+D_{\Omega }v_{1}+V_{2}diag[d_{\Omega }^{+M}]\Theta^{+M}+V_{3}D_{\Omega }\Theta +D_{\Omega }V_{4}D_{\Omega }\Theta \right)}^{T}z]+const. \end{array}$$ (7)

The *MT×MT* matrices **U**_{0…2}_ separate product terms that do not involve *ϕ*(**z**) (**U**_0_), involve multiplication by *ϕ*(**z**) only from the left-hand or right-hand side (**U**_1_), or from both sides (**U**_2_). Likewise, for the terms linear in **z**, vector and matrix terms were separated that involved *z*_*mt*_ or *θ*_*m*_ conditional on *z*_*mt*_ > *θ*_*m*_ (please see the provided MatLab code for the exact composition of these matrices). For now, the important point is that we have 2^*M× T*^ different quadratic equations, depending on the bits on and off in the binary vector **d**_Ω_. Consequently, to obtain the MAP estimator for **z**, in theory, one may consider all 2^*M×T*^ different settings for **d**_Ω_, for each solve the linear equations implied by $\partial Q_{\Omega }^{*}(Z)/\partial Z=0$, and select among those for which the solution z_*_ is consistent with the considered set Ω (if one exists; see below) the one which produces the largest value $Q_{\Omega }^{*}(z_{*})$.

In practice, this is generally not feasible. Various solution methods for piecewise linear equations have been suggested in the mathematical programming literature in the past [107; 108]. For instance, some piecewise linear problems may be recast as a linear complementarity problem [109], but the pivoting methods often used to solve it work (numerically) well only for smaller scale settings [49]. Here we therefore settled on a similar, simple Newton-type iteration scheme as proposed in [49]. Specifically, if we denote by z_*_(Ω) the solution to Eq 7 obtained with the set of constraints Ω active, the present scheme initializes with a random drawing of the {*z*_*mt*_}, sets the components of **d**_Ω_ for which *z*_*mt*_ > *θ*_*m*_ to 1 and all others to 0, and then keeps on alternating between (1) solving $\partial Q_{\Omega }^{*}(Z)/\partial Z=0$ for z_*_(Ω) and (2) flipping the bits in **d**_Ω_ for which $\mathrm{sgn}[2d_{\Omega }^{\left(k\right)}-1]\neq \mathrm{sgn}[z_{*k}(\Omega )-\theta_{k}]$, that is, for which the components of the vector

$$c≔{\left(2d_{\Omega }-1\right)}^{T}\circ (\theta -z_{*}(\Omega ))$$ (8)

are positive, until the solution to $\partial Q_{\Omega }^{*}(Z)/\partial Z=0$ is consistent with set Ω (i.e., **c** ≤ **0**).

For the problem as formulated in Brugnano & Casulli [49], these authors proved that such a solution always exists, and that the algorithm will always terminate after a finite (usually low) number of steps, given certain assumptions and provided the matrix that multiplies with the states **z** in $\partial Q_{\Omega }^{*}(Z)/\partial Z=0$ (i.e., the Hessian of $Q_{\Omega }^{*}(z_{*})$), fulfills certain conditions (Stieltjes-type; see [49] for details). This will usually *not* be the case for the present system; although the Hessian of $Q_{\Omega }^{*}(z_{*})$ will be symmetric and positive-definite (with proper parameter settings), its off-diagonal elements may be either larger or smaller than 0. Moreover, for the problem considered here, all elements of the Hessian in (7) depend on Ω, while in [49] this is only the case for the on-diagonal elements (i.e., in [49] **D**_Ω_ enters the Hessian only in additive, not multiplicative form as here). For these reasons, the Newton-type algorithm outlined above may not always converge to an exact solution (if one exists in this case) but may eventually cycle among non-solution configurations, or may not even always increase *Q*(**Z**) (i.e., Eq 5!). To bypass this, the algorithm was always terminated if one of the following three conditions was met: (i) A solution to $\partial Q_{\Omega }^{*}(Z)/\partial Z=0$ consistent with Ω was encountered; (ii) a previously probed set Ω was revisited; (iii) the constraint violation error defined by ‖**c**_+_‖_1_, the *l*_1_ norm of the positive part of **c** defined in Eq 8, went up beyond a pre-specified tolerance level (this is essentially a fast proxy for assessing the likelihood, intended to speed up iterations by using quantities already computed). With these modifications, we found that the algorithm would usually terminate after only a few iterations (<10 for the examined toy examples) and yield approximate solutions with only a few constraints still violated (<3% for the toy examples). As a caveat, unless condition (i) is met, this procedure implies that the returned solution may not even be locally optimal (in the strict mathematical sense–it would still be expected to live within an ‘elevated’ region of the optimization landscape defined by *Q*(**Z**)). On the other hand, since *Q*(**Z**) cannot keep on increasing along a closed cycle (it must ‘come back’), cycling implies there must be local maxima (or potentially saddles) located on the rims that separate different linear subspaces defined by **d**_Ω_. Hence, for the elements *k* of **z** for which the constraints are still violated in the end, that is for which *c*_*k*_ > 0 in Eq 8, one may explicitly enforce the constraints by setting the violating states **z**_{*k*}_ = **θ**_{*k*}_, then solve again for the remaining states **z**_{*l* ≠ *k*}_ (placing the solution on a ridge; or a quadratic program may be solved for the last step). Either way, it was found that even these approximate (and potentially not even locally optimal) solutions were generally (for the problems studied) in sufficiently good agreement with E(**Z**|**X**).

In the case of full EM iterations (with the parameters unknown as well), it appeared that flipping violated constraints in **d**_Ω_ one by one may often (for the scenarios studied here) improve overall performance, in the sense of yielding higher-likelihood solutions and less numerical problems (although it may leave more constraints violated in the end). Hence, this scheme was adopted here for the full EM, that is only the single bit k^*^ corresponding to the maximum element of vector **c** in Eq 8 was inverted on each iteration (the one with the largest wrong-side deviation from **θ**). In general, however, the resultant slow-down in the algorithm may not always be worth the performance gains; or a mixture of methods, with $d_{k*}^{l+1}=1-d_{k*}^{l}\;\text{with}\;k^{*}≔\arg\;\max_{k}\{c_{k}>0\}$ early on, and $d_{\left\{k\right\}}^{l+1}=1-d_{\left\{k\right\}}^{l}\forall k:c_{k}>0$ during later iterations, may be considered.

Once a (local) maximum **z**^max^ (or approximation thereof) has been obtained, the covariances may be read off from the inverse negative Hessian at **z**^max^, i.e. the elements of

$$V≔{\left(U_{0}+D_{\Omega }U_{1}+U_{1}^{T}D_{\Omega }+D_{\Omega }U_{2}D_{\Omega }\right)}^{-1}.$$ (9)

Note that this is a *local* estimate around the current maximizer **z**^max^ (i.e., oblivious to the discontinuities at the borders of the linear subspaces defined by **d**_Ω_). We then use these covariance estimates to obtain (estimates of) E[*ϕ*(**z**)], E[**z***ϕ*(**z**)^*T*^], and E[*ϕ*(**z**)*ϕ*(**z**)^*T*^], as required for the maximization step. Denoting by $F(\lambda ;\mu ,\sigma^{2})≔\int_{\lambda }^{\infty }N(x;\mu ,\sigma^{2})dx$ the complementary cumulative Gaussian, to ease subsequent derivations, let us introduce the following notation:

$$N_{k}≔N(\theta_{k};z_{k}^{\max},\sigma_{k}^{2}),\;F_{k}≔F(\theta_{k};z_{k}^{\max},\sigma_{k}^{2}),\;\sigma_{kl}^{2}≔\mathrm{cov}(z_{k}^{\max},z_{l}^{\max})\approx v_{kl}.$$ (10)

The elements of the expectancy vectors and matrices above are computed as

$$\begin{array}{l} E[\phi (z_{k})]=\sigma_{k}^{2}N_{k}+(z_{k}^{\max}-\theta_{k})F_{k},\\ E[\phi {\left(z_{k}\right)}^{2}]=({\left[z_{k}^{\max}\right]}^{2}+\sigma_{k}^{2}+\theta_{k}^{2}-2\theta_{k}z_{k}^{\max})F_{k}+(z_{k}^{\max}-\theta_{k})\sigma_{k}^{2}N_{k},\\ E[z_{k}\phi (z_{l})]=(\sigma_{kl}^{2}-\theta_{l}z_{k}^{\max}+z_{k}^{\max}z_{l}^{\max})F_{l}+z_{k}^{\max}\sigma_{l}^{2}N_{l}. \end{array}$$ (11)

The terms E[*ϕ*(*z*_*k*_)*ϕ*(*z*_*l*_)], for *k* ≠ *l*, are more tedious, and cannot be (to my knowledge and insight) computed exactly (analytically), so we develop them in a bit more detail here:

$$\begin{array}{l} E[\phi (z_{k})\phi (z_{l})]=\int_{\theta_{k}}^{\infty }\int_{\theta_{l}}^{\infty }p(z_{k},z_{l})(z_{k}-\theta_{k})(z_{l}-\theta_{l})dz_{k}dz_{l}\\ \;=\int_{\theta_{k}}^{\infty }\int_{\theta_{l}}^{\infty }p(z_{k},z_{l})z_{k}z_{l}dz_{k}dz_{l}-\theta_{k}\int_{\theta_{k}}^{\infty }\int_{\theta_{l}}^{\infty }p(z_{k},z_{l})z_{l}dz_{k}dz_{l}-\theta_{l}\int_{\theta_{k}}^{\infty }\int_{\theta_{l}}^{\infty }p(z_{k},z_{l})z_{k}dz_{k}dz_{l}\\ \;+\theta_{k}\theta_{l}\int_{\theta_{k}}^{\infty }\int_{\theta_{l}}^{\infty }p(z_{k},z_{l})dz_{k}dz_{l} \end{array}$$ (12)

The last term is just a (complementary) cumulative bivariate Gaussian evaluated with parameters specified through the approximate MAP solution (**z**^max^, **V**) (and multiplied by the thresholds). The first term we may rewrite as follows:

$$\begin{array}{l} \int_{\theta_{k}}^{\infty }\int_{\theta_{l}}^{\infty }p(z_{k},z_{l})z_{k}z_{l}dz_{k}dz_{l}=\int_{\theta_{k}}^{\infty }p(z_{k})z_{k}\int_{\theta_{l}}^{\infty }p(z_{l}|z_{k})z_{l}dz_{k}dz_{l}\\ \;=\int_{\theta_{k}}^{\infty }p(z_{k})z_{k}\left[N(\theta_{l};\mu_{l|k},\lambda_{l}^{-1})+\mu_{l|k}(1-\int_{-\infty }^{\theta_{l}}N(z_{l};\mu_{l|k},\lambda_{lk}^{-1})dz_{l}\right]dz_{k}\\ \text{where}\;\mu_{l|k}≔z_{l}^{\max}-\lambda_{l}^{-1}\lambda_{lk}(z_{k}-z_{k}^{\max})\\ \;\lambda_{l}≔\sigma_{l}^{2}/(\sigma_{k}^{2}\sigma_{l}^{2}-\sigma_{kl}^{4})\\ \;\lambda_{lk}≔-\sigma_{kl}^{2}/(\sigma_{k}^{2}\sigma_{l}^{2}-\sigma_{kl}^{4}) \end{array}$$ (13)

These are just standard results one can derive by the reverse chain rule for integration, with the *λ*’s the elements of the inverse bivariate (*k*,*l*)-covariance matrix. Note that if the variable *z*_*k*_ were removed from the first integrand in Eq 13, i.e. as in the second term in Eq 12, all terms in Eq 13 would just come down to uni- or bivariate Gaussians (times some factor) or a univariate Gaussian expectancy value, respectively. Noting this, one obtains for the second (and correspondingly for the third) term in Eq 12:

$$\begin{array}{l} \theta_{k}\int_{\theta_{k}}^{\infty }\int_{\theta_{l}}^{\infty }p(z_{k},z_{l})z_{l}dz_{k}dz_{l}=\theta_{k}\lambda_{k}N_{l}F(\theta_{k};\mu_{lk},\lambda_{l}^{-1})+\theta_{k}(z_{l}^{\max}F_{k}+\sigma_{kl}^{2}N_{k})F(\theta_{l};z_{l}^{\max},\lambda_{k}^{-1})\\ \;\text{with}\;\mu_{kl}≔z_{l}^{\max}+\sigma_{kl}^{2}/\sigma_{k}^{2}(\theta_{k}-z_{k}^{\max}) \end{array}$$ (14)

The problematic bit is the product term $\int_{\theta_{k}}^{\infty }p(z_{k})z_{k}\mu_{l|k}\int_{-\infty }^{\theta_{l}}N(z_{l};\mu_{l|k},\lambda_{lk}^{-1})dz_{l}dz_{k}$ in Eq 13, which we resolve by making the approximation $\mu_{l|k}\approx \mu_{l}=z_{l}^{\max}$. This way we have for the first term in Eq 12:

$$\begin{array}{l} \int_{\theta_{k}}^{\infty }\int_{\theta_{l}}^{\infty }p(z_{k},z_{l})z_{k}z_{l}dz_{k}dz_{l}\approx \lambda_{k}N_{l}\left[\lambda_{l}^{-1}N(\theta_{k};\mu_{lk},\lambda_{l}^{-1})+\mu_{lk}F(\theta_{k};\mu_{lk},\lambda_{l}^{-1})\right]\\ \;+\left[(\sigma_{k}^{2}z_{l}^{\max}-\sigma_{kl}^{2}z_{k}^{\max})N_{k}+(z_{k}^{\max}z_{l}^{\max}+\sigma_{kl}^{2})F_{k}\right]F(\theta_{l};\mu_{lk},\lambda_{k}^{-1}) \end{array}$$ (15)

Putting (13)–(15) together with the bivariate cumulative Gaussian yields an analytical approximation to Eq 12 that can be computed based on the quantities obtained from the approximate MAP estimate (**z**^max^, **V**).

### Expectation-maximization algorithm: Parameter estimation

Once we have estimates for E[**z**], E[**zz**^*T*^], E[*ϕ*(**z**)], E[**z***ϕ*(**z**)^*T*^], and E[*ϕ*(**z**)*ϕ*(**z**)^*T*^], the maximization step is standard and straightforward, so for convenience we just state the results here, using the notation

$$\begin{array}{l} E_{1,\Delta }≔\sum_{t=1}^{T-\Delta }E[\phi (z_{t})\phi {\left(z_{t}\right)}^{T}]\;,\;E_{2}≔\sum_{t=2}^{T}E[z_{t}z_{t-1}^{T}],\;E_{3,\Delta }≔\sum_{t=1+\Delta }^{T-1+\Delta }E[z_{t}z_{t}^{T}],\\ E_{4}≔\sum_{t=1}^{T-1}E[\phi (z_{t})z_{t}^{T}]\;,\;E_{5}≔\sum_{t=2}^{T}E[z_{t}\phi {\left(z_{t-1}\right)}^{T}],\\ F_{1}≔\sum_{t=1}^{T}x_{t}E[\phi {\left(z_{t}\right)}^{T}]\;,\;F_{2}≔\sum_{t=1}^{T}x_{t}x_{t}^{T}\;,\;F_{3}≔\sum_{t=2}^{T}s_{t}E[z_{t-1}^{T}],\\ F_{4}≔\sum_{t=2}^{T}s_{t}E[\phi {\left(z_{t-1}\right)}^{T}]\;,\;F_{5}≔\sum_{t=1}^{T}E[z_{t}]s_{t}^{T}\;,\;F_{6}≔\sum_{t=1}^{T}s_{t}s_{t}^{T} \end{array}$$ (16)

With these expectancy sums defined, one has

$$B=F_{1}E_{1,0}^{-1}$$ (17A)

$$\Gamma =\frac{1}{T}(F_{2}-F_{1}B^{T}-BF_{1}^{T}+BE_{1,0}^{T}B^{T})\circ I$$ (17B)

$$\mu_{0}=E[z_{1}]-s_{1}$$ (17C)

$$A=[(E_{2}-WE_{4}-F_{3})\circ I]{\left[E_{3,0}\circ I\right]}^{-1}$$ (17D)

$$\begin{array}{l} \Sigma =\frac{1}{T}[\mathrm{var}(z_{1})+\mu_{0}s_{1}^{T}+s_{1}\mu_{0}^{T}+E_{3,1}^{T}-F_{5}-F_{5}^{T}+F_{6}+(F_{3}-E_{2})A^{T}+A(F_{3}^{T}-E_{2}^{T})+AE_{3,0}^{T}A^{T}\\ \;+(F_{4}-E_{5})W^{T}+W(F_{4}^{T}-E_{5}^{T})+WE_{1,1}^{T}W^{T}+AE_{4}^{T}W^{T}+WE_{4}A^{T}]\circ I \end{array}$$ (17E)

Note that to avoid redundancy in the parameters, here we usually fixed **Σ** = **I** ⋅ 10^−2^ (for the toy models) or **Σ** = **I** (for the experimental data).

For **W**, since we assumed this matrix to have an off-diagonal structure (i.e., with zeros on the diagonal), we solve for each row of **W** separately:

$$\begin{array}{l} P^{\left(0\right)}≔{\left(E_{3,0}\circ I\right)}^{-1}E_{4}^{T}\\ P^{\left(1\right)}≔E_{5}-\left[(E_{2}-F_{3})\circ I\right]P^{\left(0\right)}-F_{4}\\ \forall m\in \{1\ldots M\}:W_{m,\{1:M\}\backslash \text{m}}=P_{m,\{1:M\}\backslash \text{m}}^{\left(1\right)}{\left({\left[E_{1,1}-E_{4,•m}P_{m•}^{\left(0\right)}\right]}_{\{1:M\}\backslash \text{m},\{1:M\}\backslash \text{m}}\right)}^{-1} \end{array}$$ (17F)

where the subscripts indicate the matrix elements to be pulled out, with the subscript dot denoting all elements of the corresponding column or row (e.g., ‘•m’ takes the *m*^th^ column of that matrix). Should matrices **Γ**, **Σ**, **W** of full form be desired, the derivations simplify a bit–in essence, the diagonal operator ‘∘**I**‘ in the equations above (except Eq 17D) would have to be omitted, and Eq 17F could be solved in full matrix form (instead of row-wise). An expression for input scaling matrix **C** (cf. Eq 1) is given by $C=(F_{5}-z_{1}s_{1}^{T}-WF_{4}^{T}-AF_{3}^{T}){\left(F_{6}-s_{1}s_{1}^{T}\right)}^{-1}$, but note that **C** would also show up in (17C)–(17F) (multiplying with **s**_*t*_ everywhere), as well as in the state inference equations; matrices **A**, **W**, and **C** would therefore need to be solved for simultaneously in this case (complicating the above expressions a bit; see provided MatLab code for full details).

Starting from a number of different random parameter initializations, the E- and M-steps are alternated until the log-likelihood ratio falls below a predefined tolerance level (while still increasing) or a preset maximum number of allowed iterations are exceeded. For reasons mentioned in the Results, sometimes it can actually happen that the log-likelihood ratio temporarily decreases, in which case the iterations are continued. If (*N*−*M*)^2^ ≥ *N* + *M*, factor analysis may be used to derive initial estimates for the latent states and observation parameters in (3) [27], although this was not attempted here. Another possibility is to improve initial estimates first through the much faster, corresponding LDS, before submitting them to full PLRNN estimation. For further implementational details see the MatLab code provided on GitHub (repository ‘PLRNNstsp’).

### Particle filter

To validate the approximations from our semi-analytical procedure developed above, a bootstrap particle filter as given in [26] was implemented. In bootstrap particle filtering, the state posterior distribution at time *t*,

$$\begin{array}{l} p_{\Xi }(z_{t}|x_{1},\ldots ,x_{t})=\frac{p_{\Xi }(x_{t}|z_{t})p_{\Xi }(z_{t}|x_{1},\ldots ,x_{t-1})}{p_{\Xi }(x_{t}|x_{1},\ldots ,x_{t-1})}\\ \;=\frac{p_{\Xi }(x_{t}|z_{t})\int_{z_{t-1}}p_{\Xi }(z_{t}|z_{t-1})p_{\Xi }(z_{t-1}|x_{1},\ldots ,x_{t-1})dz_{t-1}}{p_{\Xi }(x_{t}|x_{1},\ldots ,x_{t-1})} \end{array}$$ (18)

is numerically approximated through a set of ‘particles’ (samples) $\left\{z_{t}^{\left(1\right)},\ldots ,z_{t}^{\left(K\right)}\right\}$, drawn from *p*_Ξ_(**z**_*t*_ | **x**_1_,…,**x**_*t*−1_), together with a set of normalized weights $\left\{w_{t}^{\left(1\right)},\ldots ,w_{t}^{\left(K\right)}\right\}$, $w_{t}^{\left(r\right)}≔p_{\Xi }(x_{t}|z_{t}^{\left(r\right)}){\left(\sum_{k=1}^{K}p_{\Xi }(x_{t}|z_{t}^{\left(k\right)})\right)}^{-1}$. Based on this representation, moments of *p*_Ξ_(**z**_*t*_ | **x**_1:*t*_) and *p*_Ξ_(*ϕ*(**z**_*t*_) | **x**_1:*t*_) can be easily obtained by evaluating *ϕ* (or any other function of **z**) on the set of samples $\left\{z_{t}^{\left(r\right)}\right\}$ and summing the outcomes weighted with their respective normalized observation likelihoods $\left\{w_{t}^{\left(r\right)}\right\}$. A new set of samples $\left\{z_{t+1}^{\left(r\right)}\right\}$ for *t*+1 is then generated by first drawing *K* times from $\left\{z_{t}^{\left(k\right)}\right\}$ with replacement according to the weights $\left\{w_{t}^{\left(k\right)}\right\}$, and then drawing *K* new samples according to the transition probabilities $p_{\Xi }(z_{t+1}^{\left(k\right)}|z_{t}^{\left(k\right)})$ (thus approximating the integral in Eq 18). Here we used *K* = 10^4^ samples. Note that this numerical sampling scheme, like a Kalman filter, but unlike the procedure outlined above, only implements the filtering step (i.e., yields *p*_Ξ_(**z**_*t*_ | **x**_1:*t*_), not *p*_Ξ_(**z**_*t*_ | **x**_1:*T*_)). On the other hand, it gives (weakly) consistent (asymptotically unbiased; [110; 111]) estimates of all expectancies across this distribution, that is, it does not rely on the type of approximations and locally optimal solutions of our semi-analytical approach that almost inevitably will come with some bias (since, among other factors, the local or approximate mode would usually deviate from the mean by some amount for the present model).

### Experimental data sets

Details of the experimental task and electrophysiological data sets used here could be found in [41, 112]. Briefly, rats had to alternate between left and right lever presses in a Skinner box to obtain a food reward dispensed on correct choices, with a ≥ 10 s delay enforced between consecutive lever presses. While the levers were located on one side of the Skinner box, animals had to perform a nosepoke on the opposite side of the box in between lever presses for initiating the delay period, to discourage them from developing an external coding strategy (e.g., through maintenance of body posture during the delay). While animals were performing the task, multiple single units were recorded with a set of 16 tetrodes implanted bilaterally into the anterior cingulate cortex (ACC, a subdivision of rat prefrontal cortex). For the present analyses, a data set from only one of the four rats recorded on this task was selected for the present exemplary purposes, namely the one where the clearest single unit traces of delay activity were observed in the first place. This data set consisted of 30 simultaneously recorded units, of which the 19 units with spiking rates >1 Hz were retained, on 14 correct trials (only correct response trials were analyzed). The trials had variable length, but were all cut down to the same length of 14 s, including 2 s of pre-nosepoke, 5 s extending into the delay from the nosepoke, 5 s preceding the next lever press, and 2 s of post-response phase (note that this may imply temporal gaps in the middle of the delay on some trials, which were ignored here for convenience). All spike trains were convolved with Gaussian kernels (see, e.g., [12; 57; 112]), with the kernel standard deviation set individually for each unit to one half of its mean interspike-interval. Note that this also brings the observed series into tighter agreement with the Gaussian assumptions of the observation model, Eq 3. Finally, the spike time series were binned into 500 ms bins (corresponding roughly to the inverse of the overall (across all 30 recorded cells) average neural firing rates of ≈2.2 Hz), which resulted in 14 trials of 28 time bins each submitted to the estimation process. As indicated in the section ‘*State space model*’, a trial-unique initial state mean **μ**_*k*_, *k* = 1…14, was assumed for each of the 14 temporally segregated trials.
